# Trends in Ethnic Disparities in Stroke Care and Long-Term Outcomes

**DOI:** 10.1001/jamanetworkopen.2024.53252

**Published:** 2025-01-09

**Authors:** Eva S. Emmett, Matthew D. L. O’Connell, Ruonan Pei, Abdel Douiri, David Wyatt, Ajay Bhalla, Charles D. A. Wolfe, Iain J. Marshall

**Affiliations:** 1 School of Life Course and Population Sciences, King’s College London, London, United Kingdom; 2 National Institute for Health and Care Research Applied Research Collaboration South London, London, United Kingdom; 3 Department of Ageing and Health, Guy’s and St Thomas’ National Health Service Foundation Trust, London, United Kingdom

## Abstract

**Importance:**

Reducing the burden of stroke is a public health priority. While higher stroke incidence among ethnic minority populations (defined in the context of this study as individuals who are not White) is well established, reports on ethnic inequalities in care or outcomes are conflicting and often limited to hospital-admitted patients and short-term outcomes.

**Objective:**

To investigate ethnic differences in stroke care and outcomes up to 5 years after stroke and describe temporal trends and contributory factors.

**Design, Setting, and Participants:**

This population-based cohort study enrolled participants from a geographically defined area of London, United Kingdom, with prospective follow-ups up to 5 years after stroke. Participants were adults with incident stroke in 1995 to 2021. Data were analyzed from May 2023 to October 2024.

**Exposure:**

Self-reported ethnicity, categorized as Black African, Black Caribbean, White, or other (eg, Asian, other Black ethnicity, or multiple ethnicities).

**Main Outcomes and Measures:**

Outcomes of interest were stroke unit admission, thrombolysis rate, functional outcomes (measured using Barthel Index and Frenchay Activities Index), and survival.

**Results:**

Among 7280 patients (mean [SD] age, 69.3 [15.2] years; 3787 [52.0%] male) included, 3628 (63.2% of 3-month survivors) had 3-month follow-up data and 1951 (60.8% of 5-year survivors) had 5-year follow-up data. By ethnicity, 861 participants (11.8%) were Black African, 1089 (15.0%) were Black Caribbean, 4738 participants (65.1%) were White, and 592 participants (8.1%) identified as other ethnicity. Black African and Black Caribbean participants were younger than White participants (mean [SD] age, 59 [14] years, 68 [15] years, and 72 [14] years, respectively), with higher rates of hypertension (629 participants [75.0%], 805 participants [75.6%], and 2801 participants [61.8%], respectively), diabetes (246 participants [29.3%], 427 participants [40.2%], and 750 participants [16.5%], respectively), and body mass index greater than 25 (372 participants [69.0%], 370 participants [61.3%], and 1094 participants [51.6%], respectively). Black African and Black Caribbean participants had higher stroke unit admission rates than White participants in 1995 to 2003 (66 participants [42.6%], 129 participants [42.0%], and 573 participants [29.5%], respectively) but not thereafter. Black Caribbean participants had persistently lower thrombolysis rates (adjusted odds ratio compared with White participants, 0.56 [95% CI, 0.40-0.80]) and later hospital arrivals (arrival >4 hours after stroke onset: 217 Black African participants [53.8%]; 251 Black Caribbean participants [60.0%]; 654 White participants [51.2%]; *P* = .02). Black African and Black Caribbean participants had better survival than White participants (Black African participants: adjusted hazard ratio, 0.64 [95% CI, 0.54-0.77]; Black Caribbean participants: adjusted hazard ratio, 0.83 [95% CI, 0.74-0.94]) but poorer functional outcomes up to 5 years after stroke, with no significant changes over time.

**Conclusions and Relevance:**

This cohort study found major and persistent ethnic inequalities in stroke care and outcomes, and these disparities were not fully explained by sociodemographic or stroke-related factors or the high vascular risk factor prevalence in Black African and Black Caribbean participants. Drivers of poor functional outcomes require further research, but cardiovascular health–checks should be considered for Black African individuals at younger ages, and late hospital arrivals and low thrombolysis rates in Black Caribbean individuals might be amenable to tailored health campaigns.

## Key Points

**Question:**

Are there differences in stroke care and outcomes associated with ethnicity in an ethnically diverse area of London, UK, and are these differences attributable to contextual factors?

**Findings:**

In this cohort study including 7280 participants with stroke, Black Caribbean participants had lower thrombolysis rates, possibly associated with late hospital arrivals. Black African and Caribbean individuals experienced longer poststroke survival but poorer functional outcomes, and these differences were not fully explained by sociodemographic or stroke-related factors or the high vascular risk factor prevalence in Black African and Caribbean participants.

**Meaning:**

These findings suggest that addressing the high vascular risk factor burden of Black African and Caribbean patients, tailored health campaigns, and further research into sociocultural factors might help reduce persistent ethnic health inequalities.

## Teaser

This cohort study investigates ethnic differences in stroke care and outcomes up to 5 years as well as temporal trends, and contributory factors

## Introduction

Reports of racial and ethnic inequalities in stroke care and outcomes have been conflicting. We previously reported higher stroke unit admission rates in Black patients,^1^ while other studies reported ethnic minority patients (defined in the context of this study as individuals who are not White, including Black African, Black Caribbean, and individuals who identify as another ethnicity [eg, Asian, other Black ethnicity, or multiple ethnicities]) were less likely to be admitted to stroke units.^2,3^ Several studies reported lower thrombolysis or thrombectomy rates for racial and ethnic minority patients,^4,5,6,7,8,9^ while others found a higher or similar likelihood of evidence-based acute care.^3,10,11^

Higher stroke-related mortality in racial and ethnic minority patients has been well documented, largely driven by higher stroke incidence.^12,13^ However, several US studies investigating hospitalized patients with stroke up to 30 days after the index event, and Canadian and Danish studies with long-term follow-up have indicated either similar^14^ or better survival in racial and ethnic minority patients.^15,16,17,18,19^ Few studies have investigated functional outcomes, again typically at discharge or over short follow-up periods, pointing toward greater disability in racial and ethnic minority groups.^3,16,20^ Reports on temporal trends in inequalities, especially with regards to outcomes, are scarce,^8,17,20^ and the impact of the major improvements in stroke care over the last decades on racial and ethnic inequalities remains unclear.

Studies on this topic are often hospital-based, thereby excluding nonadmitted patients, typically those with mild or fatal strokes or other disincentives for admission, such as financial or sociocultural factors. This could lead to overestimating quality of care and nongeneralizable findings; whereas population-based studies, such as the South London Stroke Register (SLSR), benefit from low recruitment bias. While many studies have been undertaken within the US’s specific health care setting, the UK’s tax-funded health care system reduces financial barriers to seeking medical attention and adds a different perspective to the study of racial and ethnic health inequalities.

In this study, we describe differences in care and outcomes by patient ethnicity up to 5 years after stroke and trends since 1995. We investigate to what extent inequalities are explained by key characteristics, including health-related and socioeconomic variables.

## Methods

This cohort study was conducted as part of the SLSR and was approved by the NHS Health Research Authority and previously from the ethics committees of Guy’s and St Thomas’ Hospital, King’s College Hospital, Queen’s Square, and Westminster Hospital. All participants or their representatives gave informed oral or written consent. This report follows the Strengthening the Reporting of Observational Studies in Epidemiology (STROBE) reporting guideline.

### Study Design

The SLSR is a population-based cohort study of people with incident stroke since 1995, while residing in a geographically defined area of London, UK. The methods of the SLSR have been described previously.^21^ Multiple overlapping notification sources were used to enhance index event ascertainment, including hospital admissions, outpatient clinics, radiology reports, accident and emergency department records, and general practitioners. Stroke was verified by senior study clinicians (including A.B.) using the World Health Organization definition of stroke (*International Statistical Classification of Diseases and Related Health Problems, Tenth Revision *[*ICD-10*]).

### Exposure

Ethnicity was self-reported. Ethnicity was stratified per UK census categories^22^ into Black African, Black Caribbean, White, and other (eg, Asian, other Black ethnicity, and multiple ethnicities).

### Outcomes

Stroke unit admission (stroke unit, other hospital ward, or none) and thrombolysis (since 2003) were used as measures of evidence-based acute care interventions. Functional outcomes after stroke was assessed by the Barthel Index^23^ (BI; range, 0-20; <15 categorized as moderate or severe disability) and Frenchay Activities Index^24^ (FAI; range, 0-45; <16 categorized as inactive), collected at 3-month and 5-year follow-ups via face-to-face or telephone interview or postal questionnaire. Date of death was confirmed by UK’s Office for National Statistics.

### Covariates

Socioeconomic status was estimated by occupation, educational attainment (since 2004), and the Index of Multiple Deprivation (IMD),^25^ a neighborhood deprivation measure widely used in the UK, linked to each participant by postcode (eMethods in Supplement 1). Participants from areas among the less deprived three national fifths were combined (IMD ≥3) due to low numbers. Prestroke vascular risk factors (VRFs) were collected from hospital and general practice records, including hypertension (systolic blood pressure >140 mm Hg and diastolic >90 mm Hg), diabetes, myocardial infarction (MI), atrial fibrillation (AF), smoking (current, former, or never smoker), and body mass index (BMI; calculated as weight in kilograms divided by height in meters squared, with >25 categorized overweight or obese, since 2000). Prestroke disability was captured through the prestroke BI. Stroke severity was measured through the National Institute of Health Stroke Scales (NIHSS, since 2001, with <5 categorized mild; ≥5 to 20, moderate; and >20 severe), Glasgow Coma Scale (GCS; with <13 categorized moderate or severe; used as a measure of stroke severity in multivariable analyses), urinary incontinence, swallow impairment, and acute-phase BI. Stroke subtype was stratified into ischemic and hemorrhagic stroke (primary intracerebral or subarachnoid). Hospital arrival time was recorded since 2008. When analyzing the association between ethnicity and poststroke outcomes, acute care interventions were included as covariates.

### Statistical Analysis

Participants with incident stroke between 1995 and 2021 were included and stratified into 3 cohorts by stroke year (1995-2003, 2004-2012, 2013-2021). Follow-up data up to April 15, 2023, were included. Participants with no date of death were censored on April 15, 2023.

Baseline characteristics were stratified by ethnicity and cohort. Categorical variables were summarized as count (percentage) and continuous variables as mean (SD). *P* values for trends over time were calculated using the Cochran-Armitage test of trend for categorical and linear regression for continuous variables. Bivariate analyses were performed using χ^2^ test for categorial data and *t* test or Wilcoxon signed-rank test for continuous data. Five-year survival was analyzed using the Kaplan-Meier method and log-rank tests.

Based on literature review, we developed a logic model of causal pathways of ethnicity and stroke care or poststroke outcomes (eFigure 1 in Supplement 1). This informs a sequence of multivariable models, stepwise adding covariates to investigate to what extent ethnic inequalities were attributable to these covariates, ie, nonmodifiable risk factors (stroke year, age, sex), stroke type and severity (GCS), prestroke comorbidities (hypertension, AF, diabetes, MI, smoking, and disability and BI), or socioeconomic deprivation (IMD). For thrombolysis, additional models included hospital arrival time, and for poststroke outcomes, additional models included acute care interventions as covariates.

Using these models, we estimated the independent association of ethnicity with receipt of evidence-based acute care interventions or functional outcomes (logistic regression) or 5-year all-cause mortality (Cox proportional hazards regression). Proportional hazard assumption was tested using log-log plots, and, if not met, stratified analyses were carried out.

For covariates with at least 5% missing values (IMD score: 5.3%; smoking: 9.8%), missing values were treated as separate category in multivariable analyses. Missingness was significant for functional outcome data; its impact was assessed by comparing characteristics of those with and without missing values. The robustness of the main analysis (complete-case analysis) was tested through a sensitivity analysis using inverse probability weighting.

In further sensitivity analyses, interactions of ethnicity with confounding variables, such as year of stroke, age, sex, and prestroke VRFs, were explored by including interaction terms. This did not change the direction of results.

All tests were 2-tailed, and *P* < .05 was considered statistically significant. All statistical analyses were performed using Stata software version 18.0 (StataCorp). Data were analyzed from [placeholder] to [placeholder].

## Results

Between 1995 and 2021, 7469 participants were registered; of these, 189 (2.5%) had no ethnicity recorded and were excluded, leaving 7280 participants (mean [SD] age, 69.3 [15.2] years; 3787 [52.0%] male) for analysis. A total of 861 participants (11.8%) self-reported Black African ethnicity, 1089 participants (15.0%) self-reported Black Caribbean ethnicity, 4738 participants (65.1%) self-reported White ethnicity, and 592 participants (8.13%) self-reported other ethnic group. Since recording in 2008, 94.1% of Black African participants and 85.8% of Black Caribbean participants were born outside the UK, ie, first-generation immigrants. Black participants, especially Black African participants, were significantly younger at first-time stroke than White participants (mean [SD] age: Black African participants, 59 [14] years; Black Caribbean participants, 68 [15] years; White participants, 72 [14] years), and with a higher proportion living in more deprived neighborhoods (Table). Black African participants were more likely to be or have been in nonmanual occupations and have postsecondary education than White participants, while the opposite was found for Black Caribbean participants (Table). Black participants had higher rates of hypertension (629 Black African participants [75.0%]; 805 Black Caribbean participants [75.6%]; 2801 White participants [61.8%]), diabetes (246 Black African participants [29.3%]; 427 Black Caribbean participants [40.2%]; 750 White participants [16.5%]), and BMI greater than 25 (372 Black African participants [69.0%]; 370 Black Caribbean participants [61.3%]; 1094 White participants [51.6%]), but lower rates of AF, MI, and smoking (Table). Black participants, particularly Black African participants, had higher rates of hemorrhagic stroke but also milder stroke.

**Table.** Baseline Characteristics of Study Population, Overall and Stratified by Ethnicity

Characteristic	Participants, No. (%)					*P* value
	Total (N = 7280)	Black African (n = 861)	Black Caribbean (n = 1089)	White (n = 4738)	Other (n = 592)^a^	
Cohort						
1995-2003	2580 (35.4)	159 (18.5)	311 (28.6)	1967 (41.5)	143 (24.2)	<.001
2004-2012	2542 (34.9)	276 (32.1)	413 (37.9)	1641 (34.6)	212 (35.8)	
2013-2021	2158 (29.6)	426 (49.5)	365 (33.5)	1130 (23.8)	237 (40.0)	
Age, mean (SD), y	69.3 (15.2)	59.0 (14.0)	67.8 (14.5)	72.1 (14.4)	64.3 (16.2)	<.001
Sex						
Male	3787 (52.0)	482 (56.0)	576 (52.9)	2400 (50.7)	329 (55.6)	.007
Female	3493 (48.0)	379 (44.0)	513 (47.1)	2338 (49.3)	263 (44.4)	
IMD						
1	3760 (54.5)	485 (58.2)	616 (58.1)	2367 (53.3)	292 (52.0)	<.001
2	2430 (35.2)	295 (35.4)	376 (35.4)	1529 (34.4)	230 (41.0)	
≥3	706 (10.2)	53 (6.4)	69 (6.5)	545 (12.3)	39 (7.0)	
Occupational class						
Nonmanual occupation	1800 (38.4)	261 (50.2)	215 (30.4)	1195 (38.1)	129 (40.3)	<.001
Manual occupation	2887 (61.6)	259 (49.8)	493 (69.6)	1944 (61.9)	191 (59.7)	
Education^b^						
None or primary	355 (10.3)	48 (8.6)	75 (12.6)	175 (8.8)	57 (18.1)	<.001
Lower secondary	1240 (36.0)	134 (24.1)	223 (37.5)	807 (40.8)	76 (24.1)	
Upper secondary	1039 (30.2)	154 (27.6)	192 (32.3)	602 (30.4)	91 (28.9)	
Post secondary	812 (23.6)	221 (39.7)	104 (17.5)	396 (20.0)	91 (28.9)	
Living conditions						
Private alone	2321 (35.2)	206 (26.2)	354 (35.1)	1662 (39.0)	99 (18.5)	<.001
Private with others	3769 (57.2)	558 (71.1)	595 (59.0)	2206 (51.8)	410 (76.6)	
Care facility	495 (7.5)	21 (2.7)	59 (5.9)	389 (9.1)	26 (4.9)	
Vascular risk factors						
Hypertension	4611 (65.8)	629 (75.0)	805 (75.6)	2801 (61.8)	376 (65.5)	<.001
Diabetes	1622 (23.1)	246 (29.3)	427 (40.2)	750 (16.5)	199 (34.6)	<.001
Atrial fibrillation	1186 (17.0)	72 (8.6)	105 (10.0)	953 (21.1)	56 (9.8)	<.001
Myocardial infarction	773 (11.1)	53 (6.4)	92 (8.8)	564 (12.5)	64 (11.2)	<.001
Smoking, current or former	3863 (58.8)	218 (27.9)	535 (53.8)	2884 (67.5)	226 (43.5)	<.001
Prestroke BMI^c^						
18.5-25	1419 (39.6)	156 (28.9)	216 (35.8)	899 (42.4)	148 (46.7)	<.001
>25	1983 (55.4)	372 (69.0)	370 (61.3)	1094 (51.6)	147 (46.4)	
<18.5	178 (5.0)	11 (2.0)	18 (3.0)	127 (6.0)	22 (6.9)	
Prestroke BI <15	589 (8.5)	34 (4.1)	77 (7.3)	431 (9.6)	47 (8.3)	<.001
Stroke type						
Hemorrhagic	1320 (18.6)	212 (24.8)	203 (18.9)	750 (16.4)	155 (26.3)	<.001
Ischaemic	5778 (81.4)	644 (75.2)	871 (81.1)	3828 (83.6)	435 (73.7)	
NIHSS^d^						
Mild (≤5)	1926 (41.9)	280 (45.0)	333 (44.6)	1152 (40.6)	161 (41.4)	.005
Moderate (5-20)	2206 (48.0)	302 (48.6)	353 (47.3)	1366 (48.2)	185 (47.6)	
Severe (>20)	460 (10.0)	40 (6.4)	61 (8.2)	316 (11.2)	43 (11.1)	
GCS <13	1766 (25.3)	176 (21.4)	249 (23.8)	1179 (25.9)	162 (28.8)	.006
Incontinence	2770 (40.2)	236 (28.7)	392 (37.7)	1920 (43.0)	222 (39.2)	<.001
Swallow-test, fail	2104 (33.8)	160 (22.7)	262 (27.9)	1526 (37.2)	156 (32.8)	<.001
7-d BI <15	3117 (51.9)	340 (45.3)	487 (51.7)	2039 (53.3)	251 (51.5)	.001
Arrival >4 h^e^	1245 (53.5)	217 (53.8)	251 (60.0)	654 (51.2)	123 (53.9)	.02
Admission						
Other hospital ward	2271 (31.4)	205 (23.9)	294 (27.1)	1595 (33.9)	177 (30.1)	<.001
Stroke unit	4302 (59.5)	597 (69.7)	715 (66.0)	2632 (56.0)	358 (60.9)	
No admission	654 (9.0)	54 (6.3)	74 (6.8)	473 (10.1)	53 (9.0)	
Thrombolysis^f^	474 (12.4)	76 (14.3)	48 (7.6)	299 (12.8)	51 (15.2)	<.001
Functional outcomes						
3-mo BI <15	1059 (29.5)	116 (24.6)	178 (30.8)	664 (29.6)	101 (34.6)	.02
5-y BI <15	445 (22.8)	58 (20.4)	74 (23.2)	259 (21.9)	54 (32.3)	.02
3-mo FAI <16	1912 (59.3)	241 (60.0)	341 (65.8)	1151 (56.6)	179 (66.5)	<.001
5-y FAI <16	926 (48.8)	125 (46.0)	166 (53.2)	537 (46.9)	98 (57.6)	.02

Abbreviations: BI, Barthel Index; BMI, body mass index (calculated as weight in kilograms divided by height in meters squared); FAI, Frenchay Activities Index; GCS, Glasgow Coma Scale; IMD, Index of Multiple Deprivation; NIHSS, National Institutes of Health Stroke Scale.

^a^ Other ethnicity includes Asian, other Black ethnicity, or multiple ethnicities.

^b^ Education recorded since 2004.

^c^ BMI recorded since 2000.

^d^ NIHSS recorded since 2001.

^e^ Arrival time recorded since 2008.

^f^ Thrombolysis recorded since 2003.

### Acute Care Interventions

**Figure 1.**
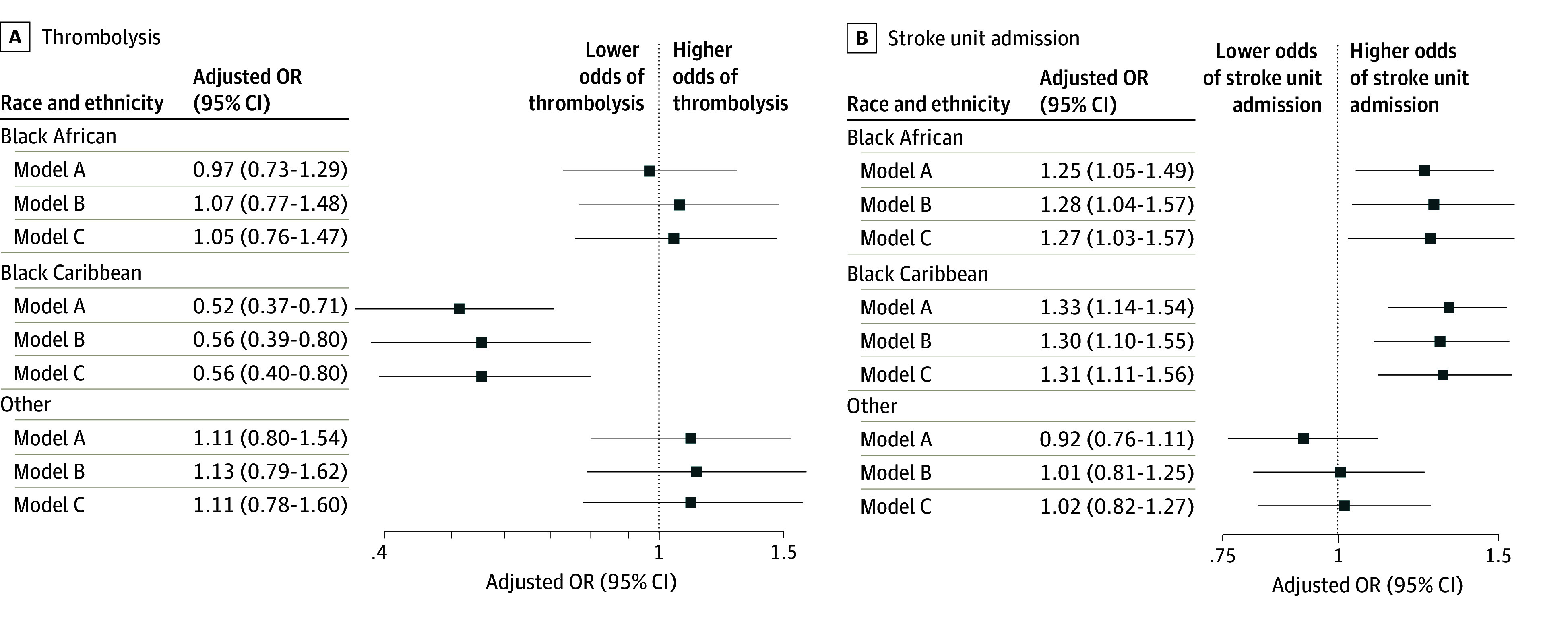
Association of Ethnicity With Thrombolysis or Stroke Unit Admission The reference group for all analyses was White patients. Thrombolysis analysis only includes participants with ischemic stroke since 2004. Model A adjusted for year of stroke, age, and sex; model B additionally adjusted for stroke severity (and stroke type for stroke unit care analysis), prestroke vascular risk factors (hypertension, atrial fibrillation, diabetes, myocardial infarction, and smoking), and Barthel Index; model C additionally adjusted for Index of Multiple Deprivation. All models and results are presented in eTable 2 and eTable 3 in Supplement 1. Other ethnicity includes Asian, other Black ethnicity, or multiple ethnicities. OR indicates odds ratio.

Black Caribbean participants had lower thrombolysis rates and were more likely than other ethnic groups to arrive at the hospital more than 4 hours after stroke onset (251 Black Caribbean participants [60.0%]; 217 Black African participants [53.8%]; 654 White participants [51.2%]; *P* = .02) (Table). Both inequalities persisted during the second and third cohort (eTable 1 in Supplement 1). Adjusting for stroke year, age, sex, stroke severity, prestroke comorbidities, and IMD did not attenuate the association between Black Caribbean ethnicity and lower thrombolysis rates compared with White ethnicity (adjusted odds ratio [aOR], 0.56 [95%CI 0.40-0.80]) (Figure 1; complete models and results are presented in eTable 2 in Supplement 1. Additional adjustment for arrival time weakened this finding so that it was no longer significant (aOR, 0.63 [95% CI, 0.39-1.01]) (eTable 2 in Supplement 1).

Black African and Black Caribbean participants were more likely to be admitted to the hospital and, if admitted, receive stroke unit care compared with White participants. Adjusting for year of stroke attenuated the association between Black African and Black Caribbean ethnicity and stroke unit care, but it was not significantly altered further by additional adjustments, including age and stroke type and severity (Black African participants: aOR, 1.27 [95% CI, 1.03-1.57]; Black Caribbean participants: aOR, 1.31 [95% CI, 1.11-1.56]) (Figure 1; complete models and results are presented in eTable 2 in Supplement 1). However, this disparity was only found in the earliest but not the more recent 2 cohorts, both in unadjusted and adjusted analyses (eTable 1 and eTable 3 in Supplement 1).

### Poststroke Outcomes

**Figure 2.**
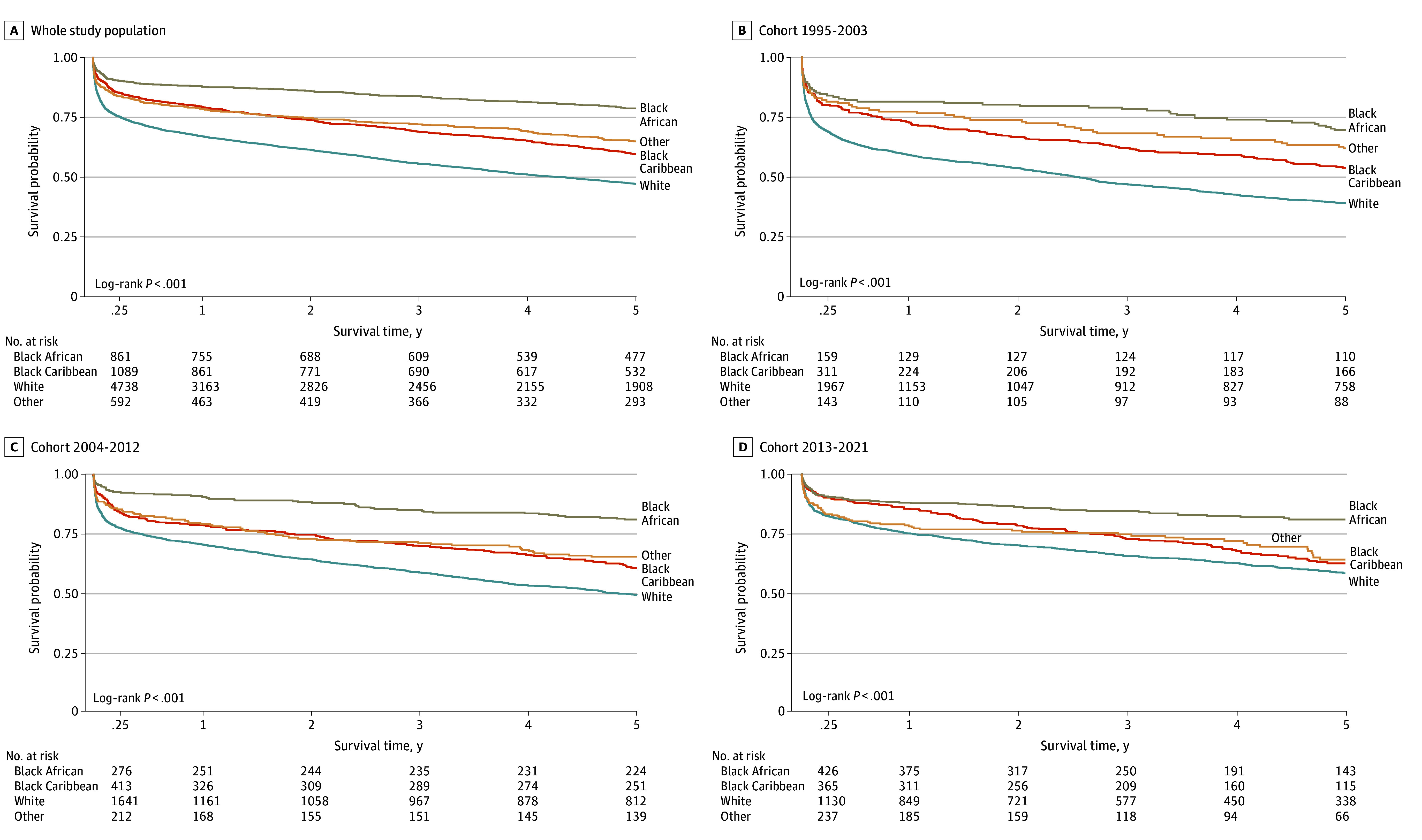
Five-Year Poststroke Survival Stratified by Cohort and Ethnicity

**Figure 3.**
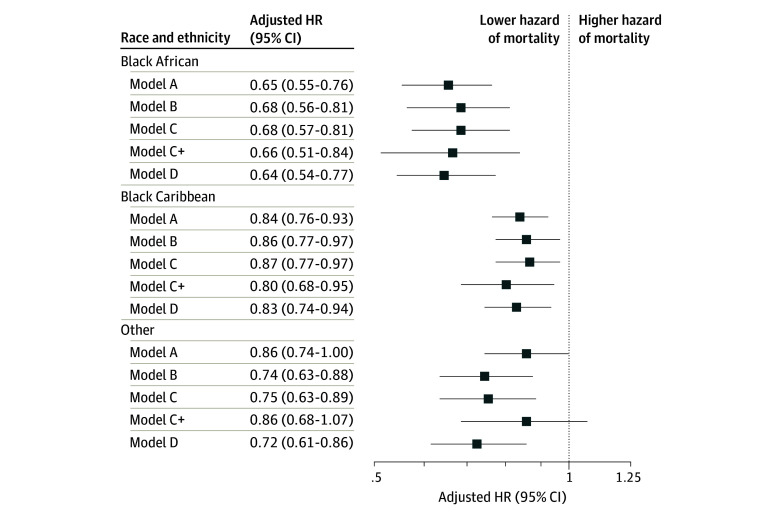
Association of Ethnicity With All-Cause Mortality Over 5 Years After Stroke For all analyses, the reference group was White patients. Model A adjusted for year of stroke, age, and sex; model B additionally adjusted for stroke severity and type, prestroke vascular risk factors (hypertension, atrial fibrillation, diabetes, myocardial infarction, and smoking), and Barthel Index; model C: additionally adjusted for stroke unit admission; model C+ additionally adjusted for thrombolysis; model D used the variables in model C and additionally adjusted for Index of Multiple Deprivation. All models/results: eTable 5 and eTable 6 in Supplement 1. Other ethnicity includes Asian, other Black ethnicity, or multiple ethnicities. HR indicates hazard ratio.

Black African participants experienced the highest and White participants experienced the lowest survival rates across all cohorts (Figure 2). Significant improvements were seen in all ethnic subgroups over time, with the largest improvement in White participants (log-rank test between cohorts, Black African: *P* = .004; Black Caribbean: *P* = .007; White: *P* < .001). Log-log plots showed that age did not follow the proportional hazards assumption, and a significant interaction term was observed between Black African ethnicity and age (eTable 4 in Supplement 1). Age-stratified analyses were carried out, showing ethnic survival disparities gradually increasing with age (eFigure 2 in Supplement 1). Adjusting for stroke year, and particularly for age (Figure 3) reduced the relative survival advantage of both Black African participants (hazard ratio [HR], 0.65 [95% CI, 0.55-0.76]) and Black Caribbean participants (HR, 0.84 [95% CI, 0.76-0.93]), but it remained significant across all Cox models (Figure 3; eTable 5 and eTable 6 in Supplement 1).

Of 5740 participants (78.9%) alive at 3 months after stroke, 3582 (62.4%) were assessed for disability and 3219 (56.0%) for inactivity, while at 5 years, of 3210 participants (44.1%) alive and having reached the 5-year follow-up time point (ie, stroke at least 5 years prior to the data cutoff on April 15, 2023), 1940 (60.4%) were assessed for disability and 1890 (58.9%) were assessed inactivity (eFigure 3 in Supplement 1). Participants with missing follow-up data were more likely to be Black African and younger, but there was no consistent association between missingness and functional outcomes (eTable 7 in Supplement 1).

**Figure 4.**
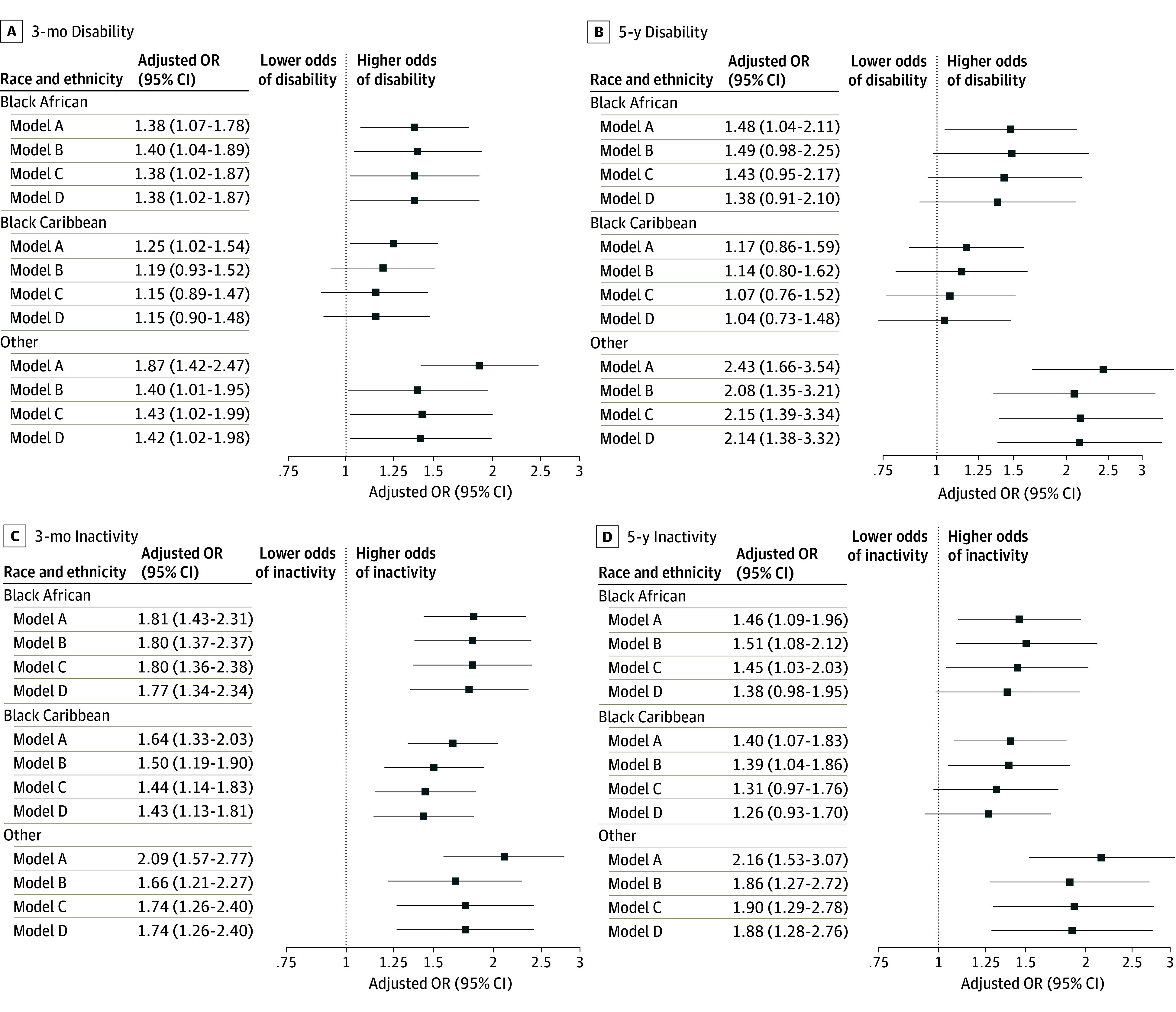
Association Between Ethnicity and Short- and Long-Term Functional Outcomes For all analyses, the reference group was White patients. Model A adjusted for year of stroke, age, and sex; model B additionally adjusted for stroke severity and type, prestroke vascular risk factors (hypertension, atrial fibrillation, diabetes, myocardial infarction, and smoking), and Barthel Index; model C additionally adjusted for stroke unit admission; model D additionally adjusted for Index of Multiple Deprivation. All models and results are presented in eTable 5 in Supplement 1. Other ethnicity includes Asian, other Black ethnicity, or multiple ethnicities.

Black Caribbean and White participants had higher proportions of disability at 3 months after stroke than Black African participants, mirroring relative disability levels before stroke (Table). There were no significant improvements in functional outcomes over time, apart from improving 3-month disability rates in the White subgroup (eTable 1 in Supplement 1). In complete case analyses, when controlling for stroke year, age, and sex (Figure 4), Black African, Black Caribbean, and other ethnicities were associated with higher levels of poststroke disability and especially inactivity. Further stepwise adjustments moderately attenuated this association. To test robustness of these results, we performed sensitivity analyses using inverse probability weighting. This approach did not alter the direction of the reported associations (eTable 8 in Supplement 1).

## Discussion

In this long-running, population-based cohort study, we found major and persistent ethnic inequalities in thrombolysis rates, survival, and poststroke functional outcomes. These were not fully explained by demographic variables, stroke type or severity, comorbidity profiles, or socioeconomic factors, indicating that further drivers, including other biological, sociocultural, psychosocial, or structural factors were likely playing a role.

Most studies investigating inequalities between Black and White people with stroke were undertaken within the US health care setting and predominantly reported lower thrombolysis rates in African American individuals.^6,7,8^ In our study, we observed persistently lower thrombolysis rates specifically in Black Caribbean but not Black African participants. Financial barriers to accessing care (eg, insurance status in the US) are unlikely to play an important role within the UK’s tax-funded health care system.

We also found a higher likelihood of delayed hospital presentation among Black Caribbean participants, as previous studies had for Black individuals.^26,27^ This might contribute to the observed lower thrombolysis rates, as indicated by the attenuated association when adjusting for arrival time. A nationwide US study additionally reported higher thrombolysis refusal rates in Black patients.^28^ Further research is required, but late arrivals and higher refusal rates, combined with our study’s indication of lower educational attainment of Black Caribbean participants, might point toward ethnic differences in the awareness of stroke requiring urgent treatment and/or cultural barriers to accessing care. Awareness campaigns could be beneficial but have been reported to disproportionally help individuals who are less disadvantaged, thereby potentially increasing health inequalities further.^29^ Future campaigns should be tailored to identify and address race- and ethnicity-specific barriers, as well as detrimental perceptions and attitudes of patients and their communities, but also health professionals.

The previously reported higher stroke unit admission rate for Black participants^1,30^ was observed only in the earliest cohort but not thereafter. It was not explained by differences in age, stroke type and severity, or comorbidities, and the reasons remain unclear. The finding of equal access to stroke unit care for ethnic minority patients since 2004 is at odds with some reports of poorer access from other countries with universal health care.^2,3^ Country-specific, structural, and race- and ethnicity-specific, or cultural factors might play a role, but the findings of this study suggest equitable stroke unit access in our setting.

Like previous studies,^15,16,17^ we found higher survival rates in Black participants compared with White participants in adjusted analyses. This finding is unexplained, and to some extent counterintuitive, as racial and ethnic minority status has been linked to socioeconomic deprivation, which was in turn reported to be associated with poorer survival after stroke.^31^ However, in this study, the link between deprivation and ethnic minority status was inconsistent among ethnic minority groups. While Black participants were more likely to live in more deprived neighborhoods, more Black African participants had postsecondary education and nonmanual occupations than White participants, potentially contributing to their survival advantage. By contrast, Black Caribbean participants had lower levels of education and more manual occupations than their peers, but still experienced better survival than their White counterparts.

Black participants, despite having more hemorrhagic strokes, had lower rates of severe strokes, AF, and MI, which were all linked to poorer survival in our analyses. Those might contribute to a survival advantage among Black patients despite our adjustments due to residual confounding, if their impact was not fully captured. Higher rates of life-sustaining interventions in Black patients with stroke were suggested in 1 US study as a possible cause for lower mortality.^32^ However, while we have no data on further acute or postacute interventions, differential rates of thrombolysis and stroke unit admission did not explain survival disparities in our study.

Besides the variables included in this study, further factors (eg, other health conditions, lifestyle, mental resilience, genetics) could contribute to the Black survival advantage. Recent ONS data for England and Wales showed generally higher life expectancies in Black individuals, which likely extend to our study cohort (Black African individuals: 84 years for men and 89 years for women; Black Caribbean individuals: 81 years for men and 85 years for women; White individuals: 80 years for men and 83 years for women).^33^ Our cohort’s Black participants were predominantly first-generation immigrants; therefore, the previously proposed healthy migrant effect^1^ may play a role. The effect likely declines with time passing since immigration as migrants adapt to the host country. This might contribute to our finding that Black African participants, who on average immigrated to the UK more recently,^34^ had better survival than Black Caribbean participants.

Like some earlier reports of short-term functional disparities,^16,20,35^ we found an association of Black ethnicity with poorer short- and long-term functional outcomes. The apparent paradox of worse functional outcome and better survival in Black patients with stroke has been described previously for short-term outcomes,^16^ but was also reported more generally for immigrant populations.^36^

Apart from lower thrombolysis rates in Black Caribbean patients, there was no persistent pattern of poorer care in ethnic minority groups. While we have no data on other aspects of care, like the quality and intensity of poststroke rehabilitation, the number of Black Caribbean participants affected by lower thrombolysis rates is small and unlikely to account for the difference in functional outcomes.

Black stroke survivors had a distinctive VRF profile, with high levels of hypertension, diabetes, and BMI greater than 25, despite their significantly younger age. As this study has no data on the severity or control rates of VRFs or new VRF diagnoses in the poststroke period (as time-varying variables), residual confounding cannot be ruled out. Higher rates and poorer control of VRFs, as well as differential rates of underdiagnosis in certain racial and ethnic groups have been reported.^5,37,38^ A specific comorbidity profile might also contribute to the divergence between survival and functional outcome by significantly affecting disability levels while having a weaker association with mortality.

Beyond comorbidities, other studies have reported poorer secondary prevention, higher recurrence rates,^39^ unhealthy lifestyles,^5^ and more cognitive impairments^40^ in Black survivors of stroke, which could all contribute to poorer poststroke functioning. The high rates of BMI greater than 25 in Black African and Black Caribbean populations might directly affect poststroke functioning and give some preliminary indication of diet and exercise, variables otherwise missing in this study. Further drivers of ethnic health inequalities have been suggested but were not available, such as individual (eg, psychological or genetic), sociocultural, and structural (eg, availability and accessibility of health or social services) factors.

### Strengths and Limitations

Important strengths of this study are its large cohort with high proportions of patients from ethnic minority groups, allowing analyses of temporal trends and subgroup analyses of Black African and Caribbean patients, showing significant inequalities between those ethnic minority groups. While most studies of differences in stroke-related outcomes between Black and White people were undertaken in the US, the UK’s universal health care coverage provides a different lens for investigating these disparities. Furthermore, this study provides long-term functional outcome data that are rarely reported.

This study also has some limitations. Due to its long-running nature, some variables were not available for the early cohort, such as NIHSS or educational attainment, and therefore were not used in multivariable analyses. Loss to follow-up led to a significant proportion of missing data on functional outcomes. Maintaining long-term contact is challenging, particularly among mobile populations. While Black African participants were more likely to have missing outcome data, there was no consistent pattern between missingness and functional outcomes after stroke. Additional sensitivity analyses using inverse probability weighting did not alter the direction of the results. Participants in the other ethnicity category experienced the highest rate of poor functional outcomes but were not analyzed further due to low numbers. These individuals should be a focus of future research.

## Conclusions

This cohort study found that significant ethnic inequalities in stroke care and outcomes in London have persisted over the last decades. They were not fully explained by demographic, health-related, or socioeconomic factors. Black African and Caribbean participants had higher rates of hypertension, diabetes, and BMI greater than 25 despite their younger age, which might warrant targeted cardiovascular health–checks in this ethnic group. Thrombolysis rates continued to be comparatively low in Black Caribbean participants, which requires further research into potential cultural barriers and tailored health campaigns. The association between Black African or Caribbean ethnicity and poorer poststroke functional outcomes might be partly explained by differential comorbidity profiles, but nonmedical, social determinants of health might also play a role.

## Notes

Supplement 1.
eMethods.
**eFigure 1.** Logic Model of Possible Causal Pathways Between Ethnicity, Stroke Care, and Outcomes **eTable 1.** Temporal Trends in Baseline Characteristics for White, Black Caribbean, and Black African Participants and All Participants Combined and for the Other Ethnic Group **eTable 2.** Association Between Ethnicity on Stroke Unit Admission and Thrombolysis **eTable 3.** Association Between Ethnicity on Stroke Unit Admission, by Cohort **eTable 4.** Cox Proportional Hazard Analysis of 5-Year All-Cause Mortality, With Interaction Term Between Ethnicity and Age (Model A) **eFigure 2.** Five-Year Survival Poststroke Stratified by Age and Ethnicity **eTable 5.** Association Between Ethnicity, 5-Year All-Cause Mortality and Short- and Long-Term Functional Outcomes (Complete Case Analysis) **eTable 6.** Cox Proportional Hazard Analysis of 5-Year All-Cause Mortality (Full Model) **eFigure 3.** Number and Follow-Up Status of Participants at 3 Months and 5 Years Poststroke **eTable 7.** Missing Functional Outcome Data at 3 Months Poststroke and at 5 Years Poststroke for 5-Year Survivors **eTable 8.** Association Between Ethnicity and Short- and Long-Term Functional Outcomes Using Inverse-Probability Weighting and Average Treatment Effect

Supplement 2.
Data Sharing Statement
