# Supplementary material for: Trends in Ethnic Disparities in Stroke Care and Long-Term Outcomes
Source: JAMA Netw Open. 2025 Jan 9;8(1):e2453252. doi: 10.1001/jamanetworkopen.2024.53252 (PMC11718558; doi:10.1001/jamanetworkopen.2024.53252)
Supplement: Supplement 1. — eMethods. eFigure 1. Logic Model of Possible Causal Pathways Between Ethnicity, Stroke Care, and Outcomes eTable 1. Temporal Trends in Baseline Characteristics for White, Black Caribbean, and Black African Participants and All Participants Combined and for the Other Ethnic Group eTable 2. Association Between Ethnicity on Stroke Unit Admission and Thrombolysis eTable 3. Association Between Ethnicity on Stroke Unit Admission, by Cohort eTable 4. Cox Proportional Hazard Analysis of 5-Year All-Cause Mortality, With Interaction Term Between Ethnicity and Age (Model A) eFigure 2. Five-Year Survival Poststroke Stratified by Age and Ethnicity eTable 5. Association Between Ethnicity, 5-Year All-Cause Mortality and Short- and Long-Term Functional Outcomes (Complete Case Analysis) eTable 6. Cox Proportional Hazard Analysis of 5-Year All-Cause Mortality (Full Model) eFigure 3. Number and Follow-Up Status of Participants at 3 Months and 5 Years Poststroke eTable 7. Missing Functional Outcome Data at 3 Months Poststroke and at 5 Years Poststroke for 5-Year Survivors eTable 8. Association Between Ethnicity and Short- and Long-Term Functional Outcomes Using Inverse-Probability Weighting and Average Treatment Effect [file jamanetwopen-e2453252-s001.pdf]

Supplemental Online Content

Emmett ES, O'Connell ML, Pei R, et al. Trends in ethnic disparities in stroke care and long-term outcomes. *JAMA Netw Open*. 2025;8(1):e2453252. doi:10.1001/jamanetworkopen.2024.53252

eMethods.

eFigure 1. Logic Model of Possible Causal Pathways Between Ethnicity, Stroke Care, and Outcomes

eTable 1. Temporal Trends in Baseline Characteristics for White, Black Caribbean, and Black African Participants and All Participants Combined and for the Other Ethnic Group

eTable 2. Association Between Ethnicity on Stroke Unit Admission and Thrombolysis

eTable 3. Association Between Ethnicity on Stroke Unit Admission, by Cohort

eTable 4. Cox Proportional Hazard Analysis of 5-Year All-Cause Mortality, With Interaction Term Between Ethnicity and Age (Model A)

eFigure 2. Five-Year Survival Poststroke Stratified by Age and Ethnicity

eTable 5. Association Between Ethnicity, 5-Year All-Cause Mortality and Short- and Long-Term Functional Outcomes (Complete Case Analysis)

eTable 6. Cox Proportional Hazard Analysis of 5-Year All-Cause Mortality (Full Model)

eFigure 3. Number and Follow-Up Status of Participants at 3 Months and 5 Years Poststroke

eTable 7. Missing Functional Outcome Data at 3 Months Poststroke and at 5 Years Poststroke for 5-Year Survivors

eTable 8. Association Between Ethnicity and Short- and Long-Term Functional Outcomes Using Inverse-Probability Weighting and Average Treatment Effect

This supplemental material has been provided by the authors to give readers additional information about their work.

© 2025 Emmett ES et al. *JAMA Network Open*.

eMethods

Index of Multiple Deprivation: Socioeconomic status (SES) was measured by the Index of Multiple Deprivation (IMD), an index first developed in 2000 and widely used in the UK. It is a composite measure of relative deprivation at a small-area level, called lower super output area (LSOA), containing a population of around 1,500 people. It consists of seven domains, which are weighted according to relevance: income 22.5%, employment 22.5%, education 13.5%, health 13.5%, crime 9.3%, barriers to housing and services 9.3%, and living environment 9.3%. The 32,482 LSOAs in England are ranked according to IMD score and categorized into national quintiles, with IMD 1 being the most deprived and IMD 5 the least deprived fifth. Areas' IMD scores change over time and updates were released in 2004, 2007, 2010, 2017, and 2019. For this analysis, the most recent IMD quintile linked to the patient's LSOA prior to first stroke was matched to the patient. For patients with stroke before 2004 the 2004 IMD dataset was used.

eFigure 1: Logic model of possible causal pathways between ethnicity, stroke care, and outcomes

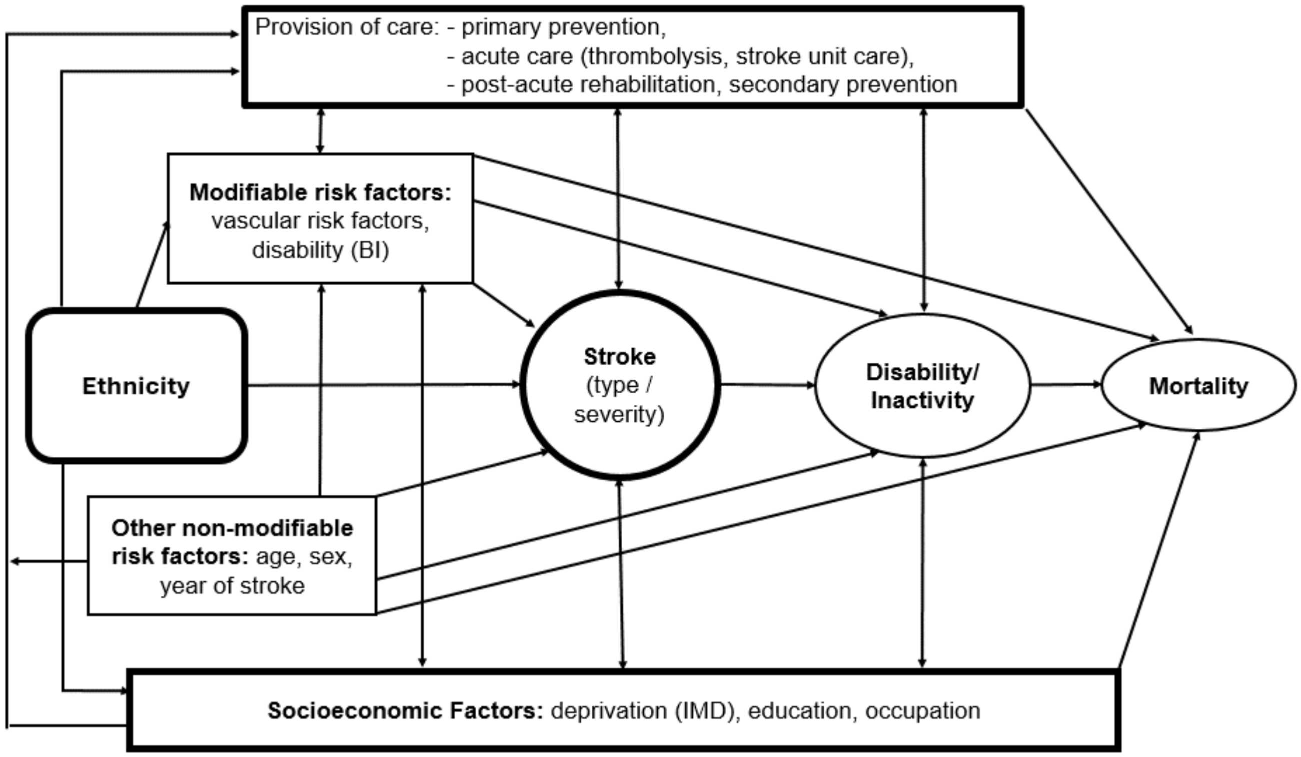

Abbreviations: BI: Barthel Index, IMD: Index of multiple deprivation

© 2025 Emmett ES et al. *JAMA Network Open*.

eTable 1a: Temporal trends in baseline characteristics for White, Black Caribbean, and Black African participants, N (%)

	WHITE				BLACK CARIBBEAN				BLACK AFRICAN			
	**1995-2003**	**2004-2012**	**2013-2021**	**p trend**	**1995-2003**	**2004-2012**	**2013-2021**	**p trend**	**1995-2003**	**2004-2012**	**2013-2021**	**p trend**
	**N=1,967**	**N=1,641**	**N=1,130**		**N=311**	**N=413**	**N=365**		**N=159**	**N=276**	**N=426**	
**Age,** mean (SD)	73.1 (13.2)	72.2 (14.9)	70.0 (15.5)	**<0.001**	66.1 (13.4)	67.3 (14.6)	70.0 (15.2)	**<0.001**	56.4 (14.3)	57.9 (13.6)	60.8 (13.9)	**<0.001**
**Female**	1,023 (52.0)	818 (49.8)	497 (44.0)	**<0.001**	144 (46.3)	194 (47.0)	175 (47.9)	**0.67**	75 (47.2)	115 (41.7)	189 (44.4)	**0.75**
**IMD**												
IMD 1	1,100 (61.2)	855 (55.9)	412 (37.0)	**<0.001**	197 (66.1)	247 (61.4)	172 (47.6)	**<0.001**	115 (75.7)	178 (68.7)	192 (45.5)	**<0.001**
IMD 2	504 (28.1)	506 (33.1)	519 (46.5)	**<0.001**	93 (31.2)	139 (34.6)	144 (39.9)	**0.019**	27 (17.8)	74 (28.6)	194 (46.0)	**<0.001**
IMD 3/4/5	192 (10.7)	169 (11.0)	184 (16.5)	**<0.001**	8 (2.7)	16 (4.0)	45 (12.5)	**<0.001**	10 (6.6)	7(2.7)	36 (8.5)	**0.10**
**Occupational class**												
Non-manual occupation	519 (32.3)	499 (41.4)	177 (54.0)	**<0.001**	43 (15.8)	117 (37.0)	55 (45.8)	**<0.001**	59 (46.5)	83(39.3)	119 (65.4)	**<0.001**
Manual occupation	1,086 (67.7)	707 (58.6)	151 (46.0)	**<0.001**	229 (84.2)	199 (63.0)	65 (54.2)	**<0.001**	68 (53.5)	128 (60.7)	63 (34.6)	**<0.001**
**Education[a]**												
None or primary	2 (7.7)	104 (9.1)	69 (8.5)	**0.68**	1 (11.1)	43 (13.8)	31 (11.3)	**0.42**	0 (0.0)	20 (9.3)	28 (8.3)	**0.81**
Lower secondary	16 (61.5)	550 (48.3)	241 (29.6)	**<0.001**	5 (55.6)	131 (42.1)	87 (31.8)	**0.005**	2 (50.0)	55 (25.6)	77 (22.8)	**0.29**
Upper secondary	4 (15.4)	320 (28.1)	278 (34.1)	**0.001**	2 (22.2)	98 (31.5)	92 (33.6)	**0.47**	1 (25.0)	60 (27.9)	93 (27.5)	**0.95**
Post secondary	4 (15.4)	165 (14.5)	227 (27.9)	**<0.001**	1 (11.1)	39 (12.5)	64 (23.4)	**<0.001**	1 (25.0)	80 (37.2)	140 (41.4)	**0.27**

(continued)

eTable 1a: Temporal trends in baseline characteristics for White, Black Caribbean, and Black African participants, N (%) (continued)

	WHITE				BLACK CARIBBEAN				BLACK AFRICAN			
	**1995-2003**	**2004-2012**	**2013-2021**		**1995-2003**	**2004-2012**	**2013-2021**		**1995-2003**	**2004-2012**	**2013-2021**	
**Living conditions**												
private alone	635 (40.5)	591 (36.8)	436 (40.3)	**0.73**	80 (31.6)	138 (33.9)	136 (39.1)	**0.051**	21 (19.3)	69 (25.7)	116 (28.5)	**0.057**
private with others	772 (49.2)	850 (52.9)	584 (54.0)	**0.011**	152 (60.1)	253 (62.2)	190 (54.6)	**0.13**	87 (79.8)	191 (71.0)	280 (68.8)	**0.039**
care facility	162 (10.3)	166 (10.3)	61 (5.6)	**0.001**	21 (8.3)	16 (3.9)	22 (6.3)	**0.42**	1 (0.9)	9 (3.3)	11 (2.7)	**0.53**
**Vascular risk factors**												
Hypertension	1,140 (62.6)	1,001 (62.2)	660 (59.9)	**0.17**	225 (76.5)	301 (73.2)	279 (77.5)	**0.71**	108 (72.5)	196 (73.1)	325 (77.0)	**0.19**
Diabetes	242 (13.2)	259 (16.0)	249 (22.6)	**<0.001**	114 (38.5)	147 (36.2)	166 (46.0)	**0.039**	27 (17.9)	75 (28.0)	144 (34.3)	**<0.001**
Atrial fibrillation	395 (21.6)	301 (18.8)	257 (23.7)	**0.36**	17 (5.9)	37 (9.2)	51 (14.4)	**<0.001**	9(5.9)	13 (4.9)	50 (12.0)	**0.003**
Myocardial infarction	247 (13.5)	159 (9.9)	158 (14.5)	**0.80**	21 (7.2)	24 (6.0)	47 (13.3)	**0.004**	8 (5.3)	11 (4.1)	34 (8.2)	**0.082**
Smoking, current or ex	1,227 (67.0)	1,007 (68.7)	650 (66.9)	**0.87**	163 (55.6)	211 (55.2)	161 (50.5)	**0.19**	40 (27.8)	85 (33.7)	93 (24.2)	**0.14**
overweight/obesity[b]	142 (46.6)	496 (53.5)	456 (51.4)	**0.39**	41 (66.1)	167 (63.7)	162 (57.9)	**0.10**	23 (67.6)	117 (69.6)	232 (68.8)	**0.99**
**pre-stroke Barthel Index<15**	148 (7.9)	141(9.2)	142 (13.1)	**<0.001**	20 (6.6)	16 (4.0)	41 (11.5)	**0.010**	1 (0.7)	6 (2.2)	27 (6.5)	**<0.001**
**Stroke type**												
Haemorrhagic stroke	332 (18.3)	227 (13.9)	191 (16.9)	**0.16**	71 (23.7)	74 (18.0)	58 (15.9)	**0.012**	53 (34.2)	57 (20.7)	102 (23.9)	**0.059**
Ischaemic stroke	1,487 (81.7)	1,403 (86.1)	938 (83.1)	**0.16**	228 (76.3)	337 (82.0)	306 (84.1)	**0.012**	102 (65.8)	218 (79.3)	324 (76.1)	**0.059**

(continued)

eTable 1a: Temporal trends in baseline characteristics for White, Black Caribbean, and Black African participants, N (%) (continued)

	WHITE				BLACK CARIBBEAN				BLACK AFRICAN			
	1995-2003	2004-2012	2013-2021		1995-2003	2004-2012	2013-2021		1995-2003	2004-2012	2013-2021	
**NIHSS[c]**												
mild, ,5	165 (33.5)	565 (38.4)	422 (48.6)	**<0.001**	35 (39.3)	158 (42.1)	140 (49.5)	**0.038**	19 (40.4)	91 (38.2)	170 (50.4)	**0.009**
moderate, 5-20	277 (56.2)	695 (47.2)	394 (45.4)	**<0.001**	49 (55.1)	176 (46.9)	128 (45.2)	**0.16**	27 (57.4)	123 (51.7)	152 (45.1)	**0.045**
severe, >20	51 (10.3)	213 (14.5)	52 (6.0)	**<0.001**	5 (5.6)	41 (10.9)	15 (5.3)	**0.23**	1 (2.1)	24 (10.1)	15 (4.5)	**0.23**
**GCS<13**	552 (29.2)	418 (26.6)	209 (19.2)	**<0.001**	72 (24.4)	102 (25.7)	75 (21.1)	**0.29**	45 (30.0)	47 (18.2)	84 (20.3)	**0.051**
**Incontinence**	881 (49.2)	662 (41.9)	377 (34.3)	**<0.001**	121 (42.6)	147 (36.5)	124 (35.0)	**0.055**	52 (35.9)	69 (26.5)	115 (27.6)	**0.13**
**Swallow-test, fail**	818 (45.9)	487 (35.1)	221 (23.7)	**<0.001**	97 (35.3)	92 (26.1)	73 (23.5)	**0.002**	52 (38.2)	45 (21.1)	63 (17.7)	**<0.001**
**7d Barthel Index<15**	855 (59.7)	712 (51.9)	472 (46.0)	**<0.001**	151 (63.2)	152 (42.1)	184 (53.8)	**0.089**	57 (52.8)	103 (42.2)	180 (45.1)	**0.38**
**Arrival >4h[d]**	NA	200 (49.4)	454 (52.1)	**NA**	NA	76 (58.0)	174 (60.8)	**NA**	NA	38 (48.7)	179 (55.1)	**NA**
**Admission**												
other hospital ward	1,054 (54.3)	305 (18.7)	236 (20.9)	**<0.001**	137 (44.6)	86 (20.9)	71(19.5)	**<0.001**	70 (45.2)	42 (15.3)	93 (21.8)	**<0.001**
stroke unit	573 (29.5)	1,220 (74.9)	839 (74.2)	**<0.001**	129 (42.0)	308 (74.9)	278 (76.2)	**<0.001**	66 (42.6)	210 (76.4)	321 (75.4)	**<0.001**
no admission	315 (16.2)	103 (6.3)	55 (4.9)	**<0.001**	41 (13.4)	17 (4.1)	16 (4.4)	**<0.001**	19 (12.3)	23 (8.4)	12 (2.8)	**<0.001**
**Thrombolysis[e]**	NA	166 (12.4)	132 (14.2)	**NA**	NA	23 (7.2)	24 (7.9)	**NA**	NA	27 (13.1)	49 (15.2)	**NA**
**Functional outcomes**												
3-month BI<15	317 (33.1)	173 (24.9)	174 (29.3)	**0.043**	65 (37.6)	42 (22.3)	71 (32.9)	**0.423**	27 (28.4)	25 (19.8)	64 (25.5)	**0.86**
5-year BI<15	102 (23.0)	112 (22.8)	45 (18.2)	**0.19**	18 (20.7)	36 (24.2)	20 (24.1)	**0.595**	5 (11.4)	34 (26.4)	19 (17.1)	**0.95**
3-month FAI<16	523 (59.1)	344 (52.0)	284 (58.2)	**0.42**	116 (74.8)	96 (54.2)	129 (69.4)	**0.396**	56 (65.1)	58 (48.3)	127 (64.8)	**0.52**
5-year FAI<16	208 (47.2)	199 (43.1)	130 (53.7)	**0.23**	50 (53.8)	65 (47.4)	51 (62.2)	**0.299**	20 (45.5)	50 (42.7)	55 (49.5)	**0.47**

Summary statistics are count (%); Percentages refer to only those with known value as denominator; p-value for trend across cohorts was calculated using Cochran-Armitage test of trend.
Abbreviations: IMD: Index of multiple deprivation, BI: Barthel Index, NIHSS: National Institutes of Health Stroke Scale, GCS: Glasgow Coma Scale, FAI: Frenchay Activities Index
a "Education" recorded since 2004, b "BMI" recorded since 2000, c "NIHSS" recorded since 2001, d "Arrival time" recorded since 2008, e "Thrombolysis" recorded since 2003

eTable 1b: Temporal trends in baseline characteristics for all study participants combined and for the “other” ethnic group, N (%)

	ALL				OTHER			
	**1995-2003**	**2004-2012**	**2013-2021**	**p trend**	**1995-2003**	**2004-2012**	**2013-2021**	**p-value**
	N=2,580	N=2,542	N=2,158		N=143	N=212	N=237	
**Ethnicity**								
White	1,967 (76.2)	1,641 (64.6)	1,130 (52.4)	**<0.001**	NA	NA	NA	NA
Black Caribbean	331 (12.1)	413 (16.3)	365 (16.9)	**<0.001**	NA	NA	NA	NA
Black African	159 (6.2)	276 (10.9)	426 (19.7)	**<0.001**	NA	NA	NA	NA
Other	143 (5.5)	212 (8.3)	237 (11.0)	**<0.001**	NA	NA	NA	NA
**Age,** mean (SD)	70.8 (14.2)	69.2 (15.5)	67.5 (15.8)	**<0.001**	64.1 (14.3)	64.4 (15.7)	64.3 (17.8)	**0.92**
**Female**	1,299 (50.3)	1,226 (48.2)	968 (44.9)	**<0.001**	57 (39.9)	99 (46.7)	107 (45.1)	**0.39**
**IMD**								
IMD 1	1,494 (62.9)	1,392 (58.3)	874 (41.0)	**<0.001**	82 (63.1)	112 (56.6)	98 (42.1)	**<0.001**
IMD 2	661 (27.8)	797 (33.4)	972 (45.6)	**<0.001**	37 (28.5)	78 (39.4)	115 (49.4)	**<0.001**
IMD 3/4/5	221 (9.3)	200 (8.4)	285 (13.4)	**<0.001**	11 (8.5)	8 (4.0)	20 (8.6)	**0.69**
**Occupational class**								
Non-manual occupation	660 (31.1)	758 (40.6)	382 (54.8)	**<0.001**	39 (33.3)	59 (43.4)	31 (46.3)	**0.06**
Manual occupation	1,461 (68.9)	1,111 (59.4)	315 (45.2)	**<0.001**	78 (66.7)	77 (56.6)	36 (53.7)	**0.06**
**Education**[a]								
None or primary	3 (7.3)	196 (10.8)	156 (9.8)	**0.47**	0 (0.0)	29 (19.5)	28 (17.1)	**0.71**
Lower secondary	23 (56.1)	772 (42.6)	445 (28.0)	**<0.001**	0 (0.0)	36 (24.2)	40 (24.4)	**0.81**
Upper secondary	8 (19.5)	526 (29.0)	505 (31.7)	**0.035**	1 (50.0)	48 (32.2)	42 (25.6)	**0.16**
Post secondary	7 (17.1)	320 (17.6)	485 (30.5)	**<0.001**	1 (50.0)	36 (24.2)	54 (32.9)	**0.13**

(continued)

eTable 1b: Temporal trends in baseline characteristics for all study participants combined and "other" ethnic group, N (%) (continued)

	ALL				OTHER			
	**1995-2003**	**2004-2012**	**2013-2021**	**p trend**	**1995-2003**	**2004-2012**	**2013-2021**	**p-value**
**Living conditions**								
private alone	749 (36.8)	833 (33.5)	739 (35.9)	**0.56**	13 (12.3)	35 (17.0)	51 (22.9)	**0.016**
private with others	1,098 (53.9)	1,456 (58.5)	1,215 (59.0)	**0.001**	87 (82.1)	162 (78.6)	161 (72.2)	**0.034**
care facility	190 (9.3)	200 (8.0)	105 (5.1)	**<0.001**	6 (5.7)	9 (4.4)	11 (4.9)	**0.85**
**Vascular risk factors**								
Hypertension	1,549 (64.7)	1,639 (65.6)	1,423 (67.2)	**0.077**	76 (58.0)	141 (67.5)	159 (67.9)	**0.079**
Diabetes	425 (17.6)	555 (22.2)	642 (30.3)	**<0.001**	42 (31.3)	74 (35.6)	83 (35.6)	**0.45**
Atrial fibrillation	426 (17.7)	370 (14.9)	390 (18.7)	**0.44**	5 (3.7)	19 (9.2)	32 (14.0)	**0.001**
Myocardial infarction	287 (11.9)	214 (8.6)	272 (13.1)	**0.30**	11 (8.2)	20 (9.6)	33 (14.3)	**0.056**
Smoking, current or ex	1,497 (62.3)	1,383 (60.3)	983 (52.5)	**<0.001**	67 (50.4)	80 (41.7)	79 (40.5)	**0.092**
overweight/obesity[b]	213 (50.4)	839 (56.7)	931 (55.5)	**0.24**	7 (31.8)	59 (48.0)	81 (47.1)	**0.42**
**pre-stroke disability (BI<15)**	174 (7.0)	179 (7.4)	236 (11.3)	**<0.001**	5 (3.6)	16 (8.0)	26 (11.3)	**0.009**
**Stroke type**								
Haemorrhagic stroke	502 (20.8)	409 (16.2)	409 (19.0)	**0.088**	46 (32.4)	51 (24.2)	58 (24.5)	**0.12**
Ischaemic stroke	1,913 (79.2)	2,118 (83.8)	1,747 (81.0)		96 (67.6)	160 (75.8)	179 (75.5)	**0.12**
**NIHSS[c]**								
mild, ,5	235 (35.0)	881 (38.8)	810 (49.2)	**<0.001**	16 (38.1)	67 (35.8)	78 (48.8)	**0.038**
moderate, 5-20	377 (56.2)	1,086 (47.8)	743 (45.1)	**<0.001**	24 (57.1)	92 (49.2)	69 (43.1)	**0.084**
severe, >20	59 (8.8)	306 (13.5)	95 (5.8)	**<0.001**	2 (4.8)	28 (15.0)	13 (8.1)	**0.613**

(continued)

eTable 1b: Temporal trends in baseline characteristics for all study participants combined and "other" ethnic group, N (%) (continued)

	ALL				OTHER			
	**1995-2003**	**2004-2012**	**2013-2021**	**p trend**	**1995-2003**	**2004-2012**	**2013-2021**	**p-value**
**GCS<13**	710 (28.7)	625 (25.8)	431 (20.6)	**<0.001**	41 (29.9)	58 (29.6)	63 (27.4)	**0.58**
**Incontinence**	1,109 (47.1)	956 (39.1)	705 (33.6)	**<0.001**	55 (41.4)	78 (38.6)	89 (38.4)	**0.61**
**Swallow-test, fail**	1,014 (43.8)	678 (32.0)	412 (23.1)	**<0.001**	47 (38.2)	54 (32.1)	55 (29.7)	**0.13**
**7d Barthel Index<15**	1,122 (59.7)	1,053 (49.1)	942 (47.5)	**<0.001**	59 (59.0)	86 (50.6)	106 (48.8)	**0.12**
**Arrival >4 hours[d]**	1 (50.0)	339 (51.1)	905 (54.5)	**0.14**		25 (51.0)	98 (54.7)	**0.64**
**Admission**								
other hospital ward	1,335 (52.5)	482 (19.1)	454 (21.0)	**<0.001**	74 (52.5)	49 (23.3)	54 (22.8)	**<0.001**
stroke unit	813 (31.9)	1,883 (74.6)	1,606 (74.4)	**<0.001**	45 (31.9)	145 (69.0)	168 (70.9)	**<0.001**
no admission	397 (15.6)	159 (6.3)	98 (4.5)	**<0.001**	22 (15.6)	16 (7.6)	15 (6.3)	**0.004**
**Thrombolysis[e]**	2 (2.8)	236 (11.7)	236 (13.6)	**0.01**	0 (0.0)	20 (13.2)	31 (17.3)	**0.20**
**Functional outcomes**								
3-month disability, BI<15	445 (34.0)	273 (24.8)	341 (29.0)	**0.0049**	36 (42.4)	33 (35.1)	32 (28.3)	**0.040**
5-year disability, BI<15	142 (22.8)	213 (25.1)	90 (18.8)	**0.17**	17 (34)	31 (39)	6 (16)	**0.10**
3-month inactivity, FAI<16	751 (62.2)	555 (52.9)	606 (62.7)	**0.91**	56 (69)	57 (63)	66 (68)	**0.92**
5-year inactivity, FAI<16	311 (49.4)	357 (44.7)	258 (54.7)	**0.15**	33 (65)	43 (52)	22 (59)	**0.53**

Summary statistics are count (%); Percentages refer to only those with known value as denominator; p-value for trend across cohorts was calculated using Cochran-Armitage test of trend.

Abbreviations: IMD: Index of multiple deprivation, BI: Barthel Index, NIHSS: National Institutes of Health Stroke Scale, GCS: Glasgow Coma Scale, FAI: Frenchay Activities Index

a "Education" recorded since 2004, b "BMI" recorded since 2000, c "NIHSS" recorded since 2001, d "Arrival time" recorded since 2008, e "Thrombolysis" recorded since 2003

eTable 2: Association between ethnicity on stroke unit admission and thrombolysis

		Black Caribbean	Black African	Other
**Stroke unit admission, OR (95%CI)**	**Adjustments**			
	unadjusted	1.53 (1.33-1.75)	1.81 (1.55-2.12)	1.22 (1.03-1.46)
	stroke year	1.26 (1.09-1.46)	1.08 (0.91-1.29)	0.89 (0.73-1.07)
	& age, sex **(Model A)**	1.33 (1.14-1.54)	1.25 (1.05-1.49)	0.92 (0.76-1.11)
	& pre-stroke VRFs, BI	1.26 (1.07-1.49)	1.18 (0.96-1.43)	0.98 (0.79-1.20)
	& stroke type and severity **(Model B)**	1.30 (1.10-1.55)	1.28 (1.04-1.57)	1.01 (0.81-1.25)
	& IMD **(Model C)**	1.31 (1.11-1.56)	1.27 (1.03-1.57)	1.02 (0.82-1.27)
**Thrombolysis[a], OR (95%CI)**				
	unadjusted	0.54 (0.39-0.75)	1.11 (0.85-1.46)	1.21 (0.88-1.67)
	stroke year	0.52 (0.38-0.72)	1.03 (0.78-1.35)	1.14 (0.83-1.58)
	& age, sex **(Model A)**	0.52 (0.37-0.71)	0.97 (0.73-1.29)	1.11 (0.80-1.54)
	& pre-stroke VRFs, BI	0.56 (0.40-0.80)	1.05 (0.76-1.45)	1.20 (0.85-1.71)
	& stroke severity **(Model B)**	0.56 (0.39-0.80)	1.07 (0.77-1.48)	1.13 (0.79-1.62)
	& IMD **(Model C)**	0.56 (0.40-0.80)	1.05 (0.76-1.47)	1.11 (0.78-1.60)
	& Arrival time	0.63 (0.39-1.01)	1.22 (0.76-1.96)	1.23 (0.71-2.13)

Odds ratio of stroke unit admission (versus other hospital ward or no admission) and thrombolysis (versus no thrombolysis; included ischemic stroke patients between 2004-2021), reference group: White ethnic group. Models stepwise adding further covariates.

Abbreviations: VRFs: vascular risk factors (hypertension, atrial fibrillation, diabetes, myocardial infarction, smoking), BI: Barthel Index, IMD: Index of Multiple Deprivation

a "Thrombolysis" recorded since 2003, only includes ischaemic stroke patients since 2004

eTable 3: Association between ethnicity on stroke unit admission, by cohort

Stroke unit admission, OR (95%CI)	Adjustments	Black Caribbean	Black African	Other
**1995-2003**	unadjusted	1.73 (1.35-2.22)	1.77 (1.27-2.47)	1.12 (0.77-1.62)
	stroke year	1.87 (1.43-2.45)	1.41 (0.99-2.01)	0.95 (0.64-1.41)
	& age, sex **(Model A)**	2.02 (1.54-2.66)	1.70 (1.17-2.48)	1.05 (0.70-1.56)
	& pre-stroke VRFs, BI	1.90 (1.14-2.58)	1.42 (0.94-2.15)	0.99 (0.64-1.51)
	& stroke type and severity **(Model B)**	2.02 (1.48-2.76)	1.51 (0.99-2.31)	1.01 (0.65-1.56)
	& IMD **(Model C)**	2.08 (1.52-2.85)	1.51.(0.98-2.31)	1.03 (0.66-1.59)
**2004-2012**	unadjusted	1.00 (0.78-1.28)	1.08 (0.80-1.46)	0.75 (0.55-1.02)
	stroke year	0.97 (0.75-1.25)	1.00 (0.74-1.36)	0.73 (0.53-1.00)
	& age, sex **(Model A)**	1.01 (0.79-1.31)	1.13 (0.83-1.55)	0.78 (0.57-1.08)
	& pre-stroke VRFs, BI	0.91 (0.69-1.20)	1.07 (0.75-1.53)	0.83 (0.58-1.19)
	& stroke severity **(Model B)**	0.99 (0.74-1.33)	1.28(0.87-1.90)	0.91 (0.62-1.33)
	& IMD **(Model C)**	1.00 (0.74-1.35)	1.28 (0.86-1.90)	0.91 (0.62-1.34)
**2004-2012**	unadjusted	1.11 (0.84-1.46)	1.06 (0.82-1.37)	0.84 (0.62-1.15)
	stroke year	1.11 (0.85-1.47)	1.10 (0.85-1.43)	0.88 (0.64-1.20)
	& age, sex **(Model A)**	1.10 (0.84-1.47)	1.28 (0.98-1.68)	0.97 (0.70-1.33)
	& pre-stroke VRFs, BI	1.11 (0.82-1.51)	1.26 (0.92-1.71)	1.06 (0.75-1.50)
	& stroke severity **(Model B)**	1.08 (0.79-1.47)	1.31 (0.95-1.81)	1.07 (0.75-1.52)
	& IMD **(Model C)**	1.10 (0.80-1.50)	1.36 (0.98-1.87)	1.10 (0.77-1.58)

Odds ratio of stroke unit admission (versus other hospital ward or no admission) and thrombolysis (versus no thrombolysis; included ischemic stroke patients between 2004-2021), reference group: White ethnic group. Models stepwise adding further covariates.

Abbreviations: VRFs: vascular risk factors (hypertension, atrial fibrillation, diabetes, myocardial infarction, smoking), BI: Barthel Index, IMD: Index of Multiple Deprivation

eTable 4: Cox proportional hazard analysis of five-year all-cause mortality, with interaction term between ethnicity and age (Model A)

	**Hazard ratio (95% CI)**
**Ethnicity, vs White**	
Black Caribbean	1.13 (0.57-2.24)
Black African	2.59 (1.19-5.61)
Other	0.78 (0.33-1.83)
**Age (years)**	1.05 (1.05-1.06)
**Ethnicity#Age**	
Black Caribbean	1.00 (0.99-1.01)
Black African	0.98 (0.97-0.99)
Other	1.00 (0.99-1.01)
Stroke year	0.97 (0.96-0.97)
Sex	1.06 (0.99-1.14)

7280 observations

eFigure 2: Five-year survival post-stroke stratified by age and ethnicity

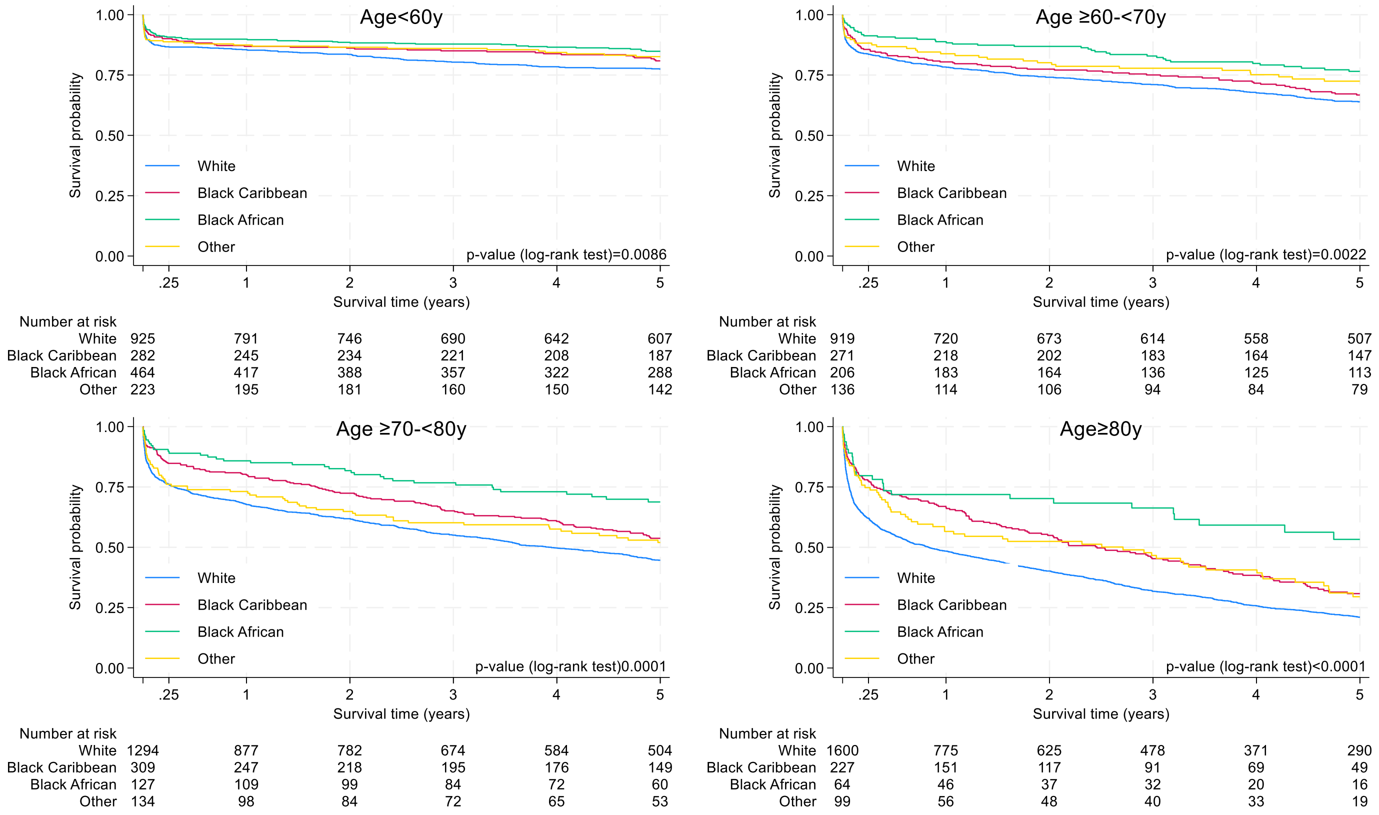

eTable 5: Association between ethnicity, 5-year all-cause mortality and short- / long-term functional outcomes (complete case analysis)

		**Black Caribbean**	**Black African**	**Other**
**Mortality, HR (95%CI)**	**Adjustments**			
	unadjusted	0.66 (0.60-0.73)	0.32 (0.27-0.37)	0.58 (0.50-0.67)
	stroke year	0.70 (0.63-0.78)	0.37 (0.32-0.43)	0.63 (0.55-0.73)
	& age, sex **(Model A)**	0.84 (0.76-0.93)	0.65 (0.55-0.76)	0.86 (0.74-1.00)
	& pre-stroke VRFs and BI	0.89 (0.80-1.00)	0.68 (0.57-0.81)	0.81 (0.69-0.95)
	& stroke type, severity **(Model B)**	0.86 (0.77-0.97)	0.68 (0.56-0.81)	0.74 (0.63-0.88)
	& stroke unit admission **(Model C)**	0.87 (0.77-0.97)	0.68 (0.57-0.81)	0.75 (0.63-0.89)
	& thrombolysis	0.80 (0.68-0.95)	0.66 (0.51-0.84)	0.86 (0.68-1.07)
	& IMD **(Model D)**	0.83 (0.74-0.94)	0.64 (0.54-0.77)	0.72 (0.61-0.86)
**3-month BI<15, OR (95%CI)**				
	unadjusted	1.06 (0.87-1.30)	0.78 (0.62-0.97)	1.26 (0.97-1.63)
	stroke year	1.09 (0.89-1.33)	0.82 (0.65-1.03)	1.30 (1.00-1.68)
	& age, sex **(Model A)**	1.25 (1.02-1.54)	1.38 (1.07-1.78)	1.87 (1.42-2.47)
	& pre-stroke VRFs and BI	1.23 (0.97-1.55)	1.45 (1.09-1.92)	1.71 (1.26-2.32)
	& stroke type, severity **(Model B)**	1.19 (0.93-1.52)	1.40 (1.04-1.89)	1.40 (1.01-1.95)
	& stroke unit admission **(Model C)**	1.15 (0.89-1.47)	1.38 (1.02-1.87)	1.43 (1.02-1.99
	& thrombolysis	1.03 (0.73-1.46)	1.32 (0.88-2.00)	0.96 (0.58-1.58)
	& IMD **(Model D)**	1.15 (0.90-1.48)	1.38 (1.02-1.87)	1.42 (1.02-1.98)
**5-year BI<15, OR (95%CI)**				
	unadjusted	1.08 (0.80-1.44)	0.91 (0.66-1.26)	1.70 (1.20-2.42)
	stroke year	1.08 (0.80-1.45)	0.93 (0.67-1.29)	1.71 (1.20-2.43)
	& age, sex **(Model A)**	1.17 (0.86-1.59)	1.48 (1.04-2.11)	2.43 (1.66-3.54)
	& pre-stroke VRFs and BI	1.21 (0.87-1.69)	1.62 (1.09-2.40)	2.33 (1.55-3.49)
	& stroke type, severity **(Model B)**	1.14 (0.80-1.62)	1.49 (0.98-2.25)	2.08 (1.35-3.21)
	& stroke unit admission **(Model C)**	1.07 (0.76-1.52)	1.43 (0.95-2.17)	2.15 (1.39-3.34)
	& thrombolysis	1.07 (0.68-1.70)	1.71 (1.03-2.86)	1.70 (0.91-3.20)
	& IMD **(Model D)**	1.04 (0.73-1.48)	1.38 (0.91-2.10)	2.14 (1.38-3.32)

(continued)

eTable 5: Association between ethnicity and 5-year all-cause mortality / short- and long-term outcomes* (continued)

		Black Caribbean	Black African	Other
**3-month FAI <16, OR (95%CI)**	**Adjustments**			
	unadjusted	1.48 (1.21-1.81)	1.15 (0.92-1.43)	1.53 (1.17-1.99)
	stroke year	1.47 (1.20-1.80)	1.13 (0.90-1.41)	1.51 (1.16-1.98)
	& age, sex **(Model A)**	1.64 (1.33-2.03)	1.81 (1.43-2.31)	2.09 (1.57-2.77)
	& pre-stroke VRFs and BI	1.55 (1.23-1.94)	1.85 (1.41-2.41)	1.91 (1.41-2.59)
	& stroke type, severity **(Model B)**	1.50 (1.19-1.90)	1.80 (1.37-2.37)	1.66 (1.21-2.27)
	& stroke unit admission **(Model C)**	1.44 (1.14-1.83)	1.80 (1.36-2.38)	1.74 (1.26-2.40)
	& thrombolysis	1.23 (0.90-1.68)	1.42 (0.99-2.04)	1.34 (0.86-2.08)
	& IMD **(Model D)**	1.43 (1.13-1.81)	1.77 (1.34-2.34)	1.74 (1.26-2.40)
**5-year FAI<16, OR (95%CI)**				
	unadjusted	1.29 (1.00-1.65)	0.96 (0.74-1.26)	1.54 (1.11-2.13)
	stroke year	1.28 (0.99-1.64)	0.93 (0.71-1.22)	1.53 (1.10-2.12)
	& age, sex **(Model A)**	1.40 (1.07-1.83)	1.46 (1.09-1.96)	2.16 (1.53-3.07)
	& pre-stroke VRFs and BI	1.45 (1.90-1.93)	1.53 (1.10-2.12)	2.03 (1.40-2.93)
	& stroke type, severity **(Model B)**	1.39 (1.04-1.86)	1.51 (1.08-2.12)	1.86 (1.27-2.72)
	& stroke unit admission **(Model C)**	1.31 (0.97-1.76)	1.45 (1.03-2.03)	1.90 (1.29-2.78)
	& thrombolysis	1.16 (0.78-1.72)	1.25 (0.81-1.91)	1.51 (0.89-2.55)
	& IMD **(Model D)**	1.26 (0.93-1.70)	1.38 (0.98-1.95)	1.88 (1.28-2.76)

Hazard ratio (HR) of mortality and Odds ratio (OR) of poor functional 3-month and 5-year outcomes versus White ethnicity. Models stepwise adding further covariates.

Abbreviations: VRFs: vascular risk factors (hypertension, atrial fibrillation, diabetes, myocardial infarction, smoking), BI: Barthel Index, FAI: Frenchay Activities Index, IMD: Index of Multiple Deprivation,

eTable 6: Cox proportional hazard analysis of five-year all-cause mortality (Model D)

	**Hazard ratio (95%CI)**
**Ethnicity, vs White**	
Black Caribbean	0.83 (0.74-0.94)
Black African	0.64 (0.54-0.77)
Other	0.72 (0.61-0.86)
**Stroke year (years)**	0.97 (0.96-0.97)
**Age (years)**	1.05 (1.04-1.05)
**Female, vs male**	0.95 (0.88-1.03)
**IMD, vs IMD1**	
IMD 2	0.87 (0.80-0.95)
IMD 3/4/5	0.60 (0.52-0.70)
unknown IMD	0.94 (0.79-1.11)
**Barthel Index<15, vs >=15**	1.45 (1.29-1.63)
**Hypertension**	0.91 (0.84-0.99)
**Atrial fibrillation**	1.33 (1.21-1.46)
**Diabetes**	1.17 (1.06-1.29)
**Myocardial infarction**	1.24 (1.09-1.37)
**Smoking,** vs never	
Current or ex	1.03 (0.95-1.13)
unknown	1.87 (1.64-2.13)
**Ischemic, vs haemorrhagic**	0.77 (0.69-0.85)
**Glasgow Coma Scale<13**	2.92 (2.68-3.18)
**Hospital admission, vs other hospital ward**	
stroke unit	0.67 (0.61-0.73)
no admission	0.54 (0.45-0.64)

Abbreviation: IMD: Index of multiple deprivation; 6217 observations

eFigure 3: Number and follow-up status of participants at 3 months and 5 years post-stroke

3-months post stroke:

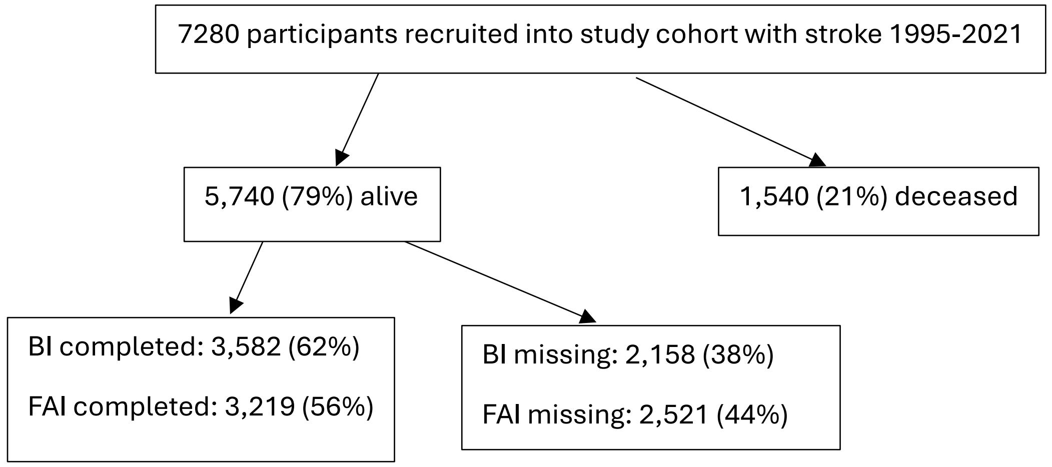

5-years post-stroke (follow-ups included up to 15 April 2023):

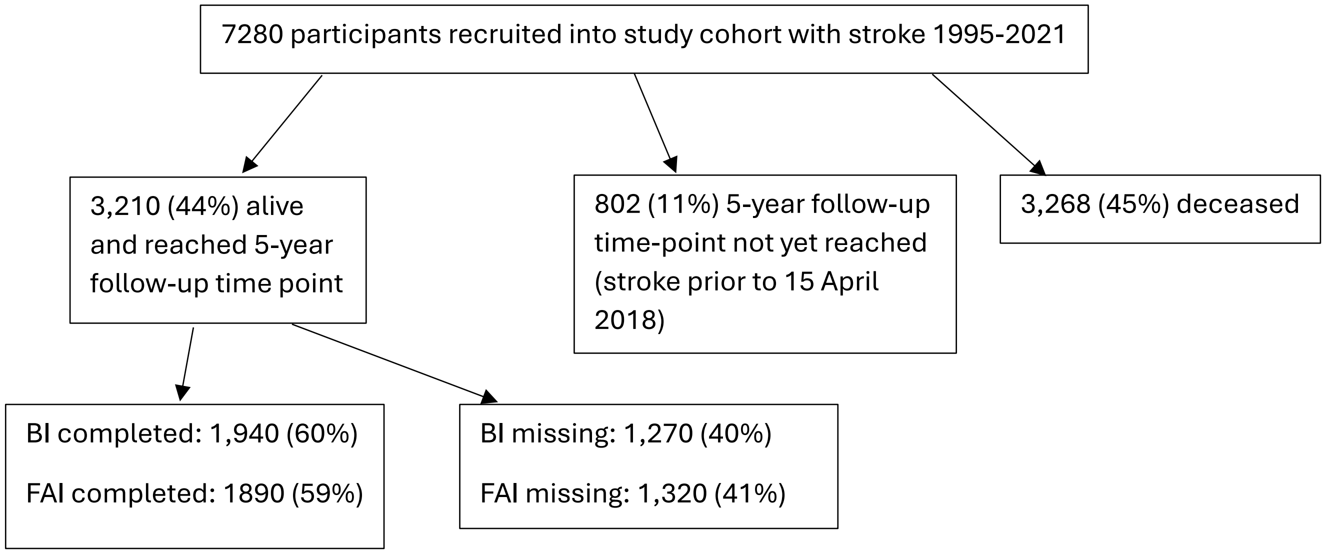

Abbreviation: BI:Barthel Index, FAI: Frenchay Activities Index

eTable 7a: Missing functional outcome data at three months post-stroke, N(%)

	3-month Barthel Index			3-month Frenchay Activities Index		
	not missing	missing		not missing	missing	
	N=3,582	N=2,158	p-value	N=3,219	N=2,521	p-value
**Ethnicity**						
White	2,242 (62.6)	1,306 (60.5)	**0.27**	2,031 (63.1)	1,517 (60.2)	**0.049**
Black Caribbean	576 (16.1)	348 (16.1)		517 (16.1)	407 (16.1)	
Black African	472 (13.2)	302 (14.0)		402 (12.5)	372 (14.8)	
Other	292 (8.2)	202 (9.4)		269 (8.4)	225 (8.9)	
**Year Group**						
1995-2003	1,308 (36.5)	533 (24.7)	**<0.001**	1,205 (37.4)	636 (25.2)	**<0.001**
2004-2012	1,101 (30.7)	957 (44.3)		1,048 (32.6)	1,010 (40.1)	
2013-2021	1,173 (32.7)	668 (31.0)		966 (30.0)	875 (34.7)	
**Age**	68.6 (14.4)	65.8 (15.8)	**<0.001**	68.8 (14.5)	65.9 (15.5)	**<0.001**
**Female**	1,679 (46.9)	954 (44.2)	**0.050**	1,518 (47.2)	1,115 (44.2)	**0.027**
**IMD**						
IMD 1	1,806 (52.7)	1,096 (53.8)	**0.10**	1,649 (53.6)	1,253 (52.4)	**0.033**
IMD 2	1,217 (35.5)	740 (36.3)		1,062 (34.5)	895 (37.4)	
IMD 3/4/5	404 (11.8)	202 (9.9)		363 (11.8)	243 (10.2)	
**Occupational class**						
Non-manual occupation	1,017 (38.7)	546 (41.0)	**0.16**	899 (37.3)	664 (42.7)	**<0.001**
Manual occupation	1,613 (61.3)	787 (59.0)		1,508 (62.7)	892 (57.3)	
**Education**[a]						
None or primary	199 (10.2)	120 (9.5)	**<0.001**	176 (10.2)	143 (9.6)	**<0.001**
Lower secondary	720 (37.0)	424 (33.6)		648 (37.5)	496 (33.5)	
Upper secondary	614 (31.5)	369 (29.2)		549 (31.8)	434 (29.3)	
Post secondary	415 (21.3)	350 (27.7)		356 (20.6)	409 (27.6)	
**Living conditions**						
private alone	1,168 (35.2)	672 (34.7)	**0.37**	1,041 (34.9)	799 (35.3)	**0.19**
private with others	1,934 (58.4)	1,155 (59.7)		1,746 (58.5)	1,343 (59.3)	
care facility	212 (6.4)	107 (5.5)		197 (6.6)	122 (5.4)	

(continued)

eTable 7a: Missing functional outcome data at three months post-stroke, N(%), continued

	3-month Barthel Index			3-month Frenchay Activities Index		
	not missing	missing		not missing	missing	
**Vascular risk factors**						
Hypertension	2,361 (67.2)	1,322 (64.1)	**0.019**	2,141 (67.8)	1,542 (63.7)	**0.001**
Diabetes	831 (23.6)	490 (23.8)	**0.83**	735 (23.2)	586 (24.3)	**0.36**
Atrial fibrillation	515 (14.7)	285 (13.9)	**0.38**	482 (15.4)	318 (13.2)	**0.024**
Myocardial infarction	367 (10.5)	189 (9.2)	**0.10**	333 (10.6)	223 (9.2)	**0.088**
Smoking, current or ex	2,052 (59.4)	1,182 (60.0)	**0.65**	1,867 (60.0)	1,367 (59.2)	**0.56**
overweight/obesity[b]	1,130 (57.0)	669 (56.0)	**0.58**	1,009 (57.3)	790 (55.8)	**0.47**
**pre-stroke BI<15**	193 (5.5)	122 (6.0)	**0.42**	177 (5.6)	138 (5.8)	**0.79**
**Stroke type**						
Haemorrhagic stroke	519 (14.6)	343 (16.1)	**0.12**	464 (14.5)	398 (16.0)	**0.12**
Ischaemic stroke	3,032 (85.4)	1,782 (83.9)		2,727 (85.5)	2,087 (84.0)	
**NIHSS[c]**						
mild,<=5	1,058 (46.4)	802 (50.8)	**0.003**	914 (44.5)	946 (52.3)	**<0.001**
moderate, 5-20	1,141 (50.0)	704 (44.6)		1,060 (51.6)	785 (43.4)	
severe, >20	83 (3.6)	73 (4.6)		79 (3.8)	77 (4.3)	
**GCS<13**	468 (13.4)	310 (15.4)	**0.043**	427 (13.6)	351 (14.8)	**0.21**
**Incontinence**	1,044 (29.9)	549 (27.1)	**0.029**	965 (30.7)	628 (26.4)	**<0.001**
**Swallow-test, fail**	766 (23.4)	373 (21.8)	**0.21**	710 (24.0)	429 (21.1)	**0.016**
**7d BI<15**	1,571 (47.9)	718 (39.2)	**<0.001**	1,459 (49.6)	830 (38.2)	**<0.001**
**Arrival >4h[d]**	697 (56.8)	424 (54.9)	**0.42**	585 (56.4)	536 (55.7)	**0.73**
**Admission**						
other hospital ward	901 (25.2)	572 (26.9)	**<0.001**	811 (25.3)	662 (26.6)	**<0.001**
stroke unit	2,373 (66.4)	1,266 (59.5)		2,132 (66.4)	1,507 (60.5)	
no admission	299 (8.4)	289 (13.6)		268 (8.3)	320 (12.9)	
**Thrombolysis[e]**	250 (13.2)	156 (11.3)	**0.11**	221 (13.2)	185 (11.6)	**0.16**
**Functional outcomes**						
3-mo BI<15	1,055 (29.5)	NA	NA	962 (30.3)	93 (23.0)	**0.002**
5-year BI<15	311 (23.6)	134 (21.3)	**0.26**	297 (23.7)	148 (21.3)	**0.22**
3-mo FAI<16	1,881 (59.2)	27 (64.3)	**0.51**	1,908 (59.3)	NA	NA
5-y FAI<16	652 (50.5)	274 (45.1)	**0.029**	622 (50.7)	304 (45.2)	**0.022**

Summary statistics are count (%); Percentages refer to only those with known value as denominator;

a “Education” recorded since 2004, b “BMI” recorded since 2000, c “NIHSS” recorded since 2001, d “Arrival time” recorded since 2008, e “Thrombolysis” recorded since 2003

Abbreviations: IMD: Index of multiple deprivation, NIHSS: National Institutes of Health Stroke Scale, GCS: Glasgow Coma Scale, OT: occupational therapy, PT: physiotherapy, SALT: speech and language-therapy, FAI: Frenchay Activities Index

eTable 7b: Missing functional outcome data at five years post-stroke, N (%), of 5-year survivors (participants with stroke prior to 15 April 2018 and alive at 5-years post-stroke)

	**5-year Barthel Index**			**5-year Frenchay Activities Index**		
	**not missing**	**missing**		**not missing**	**missing**	
	**N=1,940**	**N=1,270**	**p-value**	**N=1,890**	**N=1,320**	**p-value**
**Ethnicity**						
White	1,176 (60.6%)	732 (57.6%)	**0.34**	1,141 (60.4%)	767 (58.1%)	**0.56**
Black Caribbean	317 (16.3%)	215 (16.9%)		310 (16.4%)	222 (16.8%)	
Black African	280 (14.4%)	197 (15.5%)		269 (14.2%)	208 (15.8%)	
Other	167 (8.6%)	126 (9.9%)		170 (9.0%)	123 (9.3%)	
**Year Group**						
1995-2003	623 (32.1%)	499 (39.3%)	**<0.001**	628 (33.2%)	494 (37.4%)	**<0.001**
2004-2012	846 (43.6%)	580 (45.7%)		796 (42.1%)	630 (47.7%)	
2013-2021	471 (24.3%)	191 (15.0%)		466 (24.7%)	196 (14.8%)	
**Age**	64.1 (13.9)	61.7 (15.1)	**<0.001**	64.1 (13.9)	61.9 (15.1)	**0.001**
**Female**	847 (43.7%)	531 (41.8%)	**0.3**	821 (43.4%)	557 (42.2%)	**0.48**
**IMD**						
IMD 1	1,009 (54.6%)	671 (57.0%)	**0.36**	993 (55.2%)	687 (56.1%)	0.7
IMD 2	632 (34.2%)	374 (31.7%)		609 (33.8%)	397 (32.4%)	
IMD 3/4/5	206 (11.2%)	133 (11.3%)		198 (11.0%)	141 (11.5%)	
**Occupational class**						
Non-manual occupation	602 (40.6%)	370 (38.8%)	**0.38**	590 (40.9%)	382 (38.5%)	**0.23**
Manual occupation	880 (59.4%)	583 (61.2%)		852 (59.1%)	611 (61.5%)	
**Education**[a]						
None or primary	104 (8.9%)	64 (9.6%)	**0.34**	96 (8.5%)	72 (10.1%)	**0.48**
Lower secondary	401 (34.2%)	213 (32.1%)		383 (34.1%)	231 (32.4%)	
Upper secondary	395 (33.7%)	210 (31.6%)		377 (33.6%)	228 (31.9%)	
Post secondary	273 (23.3%)	177 (26.7%)		267 (23.8%)	183 (25.6%)	
**Living conditions**						
private alone	613 (34.7%)	368 (31.5%)	**0.17**	588 (34.2%)	393 (32.4%)	**0.25**
private with others	1,087 (61.6%)	749 (64.1%)		1,071 (62.3%)	765 (63.0%)	
care facility	66 (3.7%)	51 (4.4%)		61 (3.5%)	56 (4.6%)	

(continued)

eTable 7b: Missing functional outcome data at five years post-stroke, N (%), continued

	5-year Barthel Index			5-year Frenchay Activities Index		
	**not missing**	**missing**		**not missing**	**missing**	
**Vascular risk factors**						
Hypertension	1,227 (65.1%)	749 (61.1%)	**0.025**	1,202 (65.4%)	774 (60.8%)	**0.008**
Diabetes	395 (20.9%)	244 (20.0%)	**0.05**	386 (21.0%)	253 (19.9%)	**0.46**
Atrial fibrillation	188 (10.0%)	120 (9.8%)	**0.85**	177 (9.7%)	131 (10.3%)	**0.57**
Myocardial infarction	158 (8.4%)	96 (7.9%)	**0.6**	156 (8.5%)	98 (7.7%)	**0.43**
Smoking, current or ex	1,127 (59.8%)	749 (62.7%)	**0.11**	1,104 (60.0%)	772 (62.2%)	**0.22**
overweight/obesity[b]	672 (61.7%)	370 (57.9%)	**0.12**	642 (61.4%)	400 (58.6%)	**0.23**
**pre-stroke BI<15**	27 (1.4%)	30 (2.4%)	**0.038**	24 (1.3%)	33 (2.6%)	**0.008**
**Stroke type**						
Haemorrhagic stroke	324 (16.8%)	236 (18.9%)	**0.14**	314 (16.7%)	246 (18.9%)	**0.11**
Ischaemic stroke	1,601 (83.2%)	1,014 (81.1%)		1,562 (83.3%)	1,053 (81.1%)	
**NIHSS[c]**						
mild,<=5	704 (52.7%)	438 (50.6%)	**0.61**	674 (52.6%)	468 (50.9%)	**0.67**
moderate, 5-20	584 (43.7%)	397 (45.9%)		561 (43.8%)	420 (45.7%)	
severe, >20	47 (3.5%)	30 (3.5%)		46 (3.6%)	31 (3.4%)	
**GCS<13**	235 (12.6%)	160 (13.4%)	**0.52**	231 (12.7%)	164 (13.2%)	**0.67**
**Incontinence**	395 (21.0%)	289 (24.2%)	**0.034**	389 (21.2%)	295 (23.8%)	**0.091**
**Swallow-test, fail**	306 (18.1%)	207 (19.6%)	**0.33**	303 (18.3%)	210 (19.1%)	**0.59**
**7d BI<15**	587 (34.4%)	403 (36.8%)	**0.2**	583 (35.0%)	407 (35.8%)	**0.69**
**Arrival >4h[d]**	332 (58.0%)	198 (59.3%)	**0.71**	334 (59.2%)	196 (57.3%)	**0.57**
**Admission**						
other hospital ward	472 (24.5%)	351 (28.0%)	**0.05**	465 (24.7%)	358 (27.4%)	**0.19**
stroke unit	1,230 (63.8%)	748 (59.6%)		1,190 (63.3%)	788 (60.4%)	
no admission	227 (11.8%)	156 (12.4%)		224 (11.9%)	159 (12.2%)	
**Thrombolysis[e]**	124 (11.3%)	81 (12.8%)	**0.35**	115 (11.0%)	90 (13.3%)	**0.14**
**Functional outcomes**						
3-mo BI<15	235 (17.9%)	119 (18.8%)	**0.65**	238 (18.6%)	116 (17.6%)	**0.6**
5-year BI<15	440 (22.7%)	NA	NA	411 (22.5%)	29 (25.9%)	**0.4**
3-mo FAI<16	558 (44.9%)	278 (46.6%)	**0.47**	558 (45.8%)	278 (44.7%)	**0.65**
5-y FAI<16	882 (48.2%)	38 (61.3%)	**0.043**	920 (48.7%)	NA	NA

Summary statistics are count (%); Percentages refer to only those with known value as denominator

a “Education” recorded since 2004, b “BMI” recorded since 2000, c “NIHSS” recorded since 2001, d “Arrival time” recorded since 2008, e “Thrombolysis” recorded since 2003

Abbreviations: IMD: Index of multiple deprivation, NIHSS: National Institutes of Health Stroke Scale, GCS: Glasgow Coma Scale, OT: occupational therapy, PT: physiotherapy, SALT: speech and language-therapy, FAI: Frenchay Activities Index

eTable 8: Association between ethnicity and short- / long-term functional outcomes using inverse-probability weighting, average treatment effect, coefficient (95%CI), vs White

Outcome	Adjustments	Black Caribbean	Black African	Other
**3-month BI <15**	**Model A**	0.04 (0.00-0.08)	0.03 (-0.03-0.09)	0.13 (0.07-0.19)
	**Model B**	0.04 (-0.01-0.08)	0.04 (-0.06-0.15)	0.08 (0.01-0.15)
	**Model C**	0.04 (-0.01-0.08)	0.04 (-0.07-0.15)	0.09 (0.01-0.16)
	**Model D**	0.05 (0.00-0.09)	0.04 (-0.06-0.14)	0.09 (0.01-0.16)
**5-year BI <15**	**Model A**	0.03 (-0.02-0.07)	0.02 (-0.04-0.08)	0.15 (0.08-0.23)
	**Model B**	0.00 (-0.05-0.06)	0.02 (-0.09-0.13)	0.11 (0.02-0.20)
	**Model C**	0.00 (-0.05-0.05)	0.02 (-0.09-0.13)	0.11 (0.02-0.21)
	**Model D**	0.00 (-0.06-0.05)	0.04 (-0.08-0.16)	0.11 (0.02-0.21)
**3-month FAI <16**	**Model A**	0.12 (0.07-0.16)	0.13 (0.07-0.19)	0.15 (0.09-0.21)
	**Model B**	0.10 (0.04-0.15)	0.09 (0.01-0.18)	0.13 (0.05-0.20)
	**Model C**	0.10 (0.04-0.15)	0.09 (0.01-0.18)	0.13 (0.05-0.20)
	**Model D**	0.11 (0.05-0.16)	0.09 (0.00-0.17)	0.13 (0.05-0.20)
**5-year FAI <16**	**Model A**	0.08 (0.02-0.14)	0.07 (0.00-0.14)	0.16 (0.08-0.24)
	**Model B**	0.07 (0.00-0.14)	0.15 (0.04-0.26)	0.11 (0.01-0.20)
	**Model C**	0.06 (-0.01-0.13)	0.15 (0.04-0.26)	0.11 (0.01-0.20)
	**Model D**	0.06 (-0.02-0.13)	0.14 (0.03-0.25)	0.11 (0.01-0.20)

Model A: adjusted for stroke year, age, sex; Model B: like Model A plus additionally adjusted for stroke type/severity and pre-stroke co-morbidities (hypertension, atrial fibrillation, diabetes, myocardial infarction, smoking, Barthel Index); Model C: like Model B plus additionally adjusted for type of hospital admission; Model D: like Model C plus additionally adjusted for IMD

Abbreviations: BI: Barthel Index, FAI: Frenchay Activities Index
