# Supplementary material for: Trends in Ethnic Disparities in Stroke Care and Long-Term Outcomes
Source: JAMA Netw Open. 2025 Jan 9;8(1):e2453252. doi: 10.1001/jamanetworkopen.2024.53252 (PMC11718558; doi:10.1001/jamanetworkopen.2024.53252)
Supplement: Supplement 2. — Data Sharing Statement [file jamanetwopen-e2453252-s002.pdf]

Data Sharing Statement

Emmett. Trends in Ethnic Disparities in Stroke Care and Long-Term Outcomes. *JAMA Netw Open*. Published January 09, 2025. doi:10.1001/jamanetworkopen.2024.53252

Data
Data available: Yes
Data types: Deidentified participant data, Data dictionary
How to access data: Because of the sensitive nature of the data collected for this study, requests to access the data set for academic use should be made to the South London Stroke Register (SLSR) team:
https://www.kcl.ac.uk/lsm/research/divisions/hscr/research/groups/stroke/index.aspx
When available: With publication

Supporting Documents
Document types: None

Additional Information
Who can access the data: Researchers whose proposed use of the data has been approved
Types of analyses: for a specified purpose
Mechanisms of data availability: Data will be made available after approval of a proposal through the SLSR investigator team, including a signed data access agreement.
Any additional restrictions: NA
